# Engineering Polymers via Understanding the Effect of Anchoring Groups for Highly Stable Liquid Metal Nanoparticles

**DOI:** 10.1021/acsanm.1c04138

**Published:** 2022-02-14

**Authors:** Xumin Huang, Tianhong Xu, Ao Shen, Thomas P. Davis, Ruirui Qiao, Shi-Yang Tang

**Affiliations:** † Australian Institute for Bioengineering and Nanotechnology, The University of Queensland, Brisbane, QLD 4072, Australia; ‡ Department of Electronic, Electrical and Systems Engineering, University of Birmingham, Edgbaston, Birmingham B15 2TT, U.K.

**Keywords:** liquid metal, nanocomposites, brushed polymers, colloidal stability, biological solutions

## Abstract

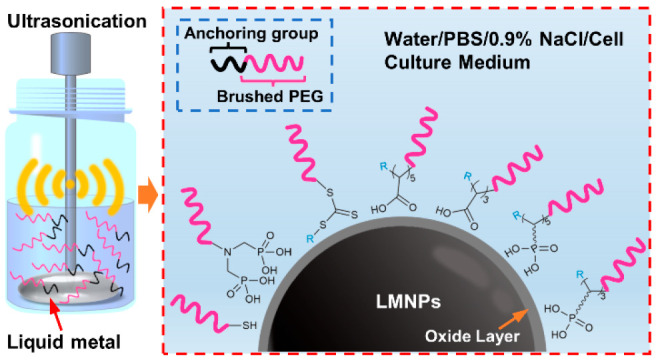

Liquid metal nanoparticles (LMNPs) have recently attracted much attention as soft functional materials for various biorelated applications. Despite the fact that several reports demonstrate highly stable LMNPs in aqueous solutions or organic solvents, it is still challenging to stabilize LMNPs in biological media with complex ionic environments. LMNPs grafted with functional polymers (polymers/LMNPs) have been fabricated for maintaining their colloidal and chemical stability; however, to the best of our knowledge, no related work has been conducted to systematically investigate the effect of anchoring groups on the stability of LMNPs. Herein, various anchoring groups, including phosphonic acids, trithiolcarbonates, thiols, and carboxylic acids, are incorporated into brush polymers via reversible addition-fragmentation chain transfer (RAFT) polymerization to graft LMNPs. Both the colloidal and chemical stability of such polymer/LMNP systems are then investigated in various biological media. Moreover, the influence of multidentate ligands is also investigated by incorporating different numbers of carboxylic or phosphonic acid into the brush polymers. We discover that increasing the number of anchoring groups enhances the colloidal stability of LMNPs, while polymers bearing phosphonic acids provide the optimum chemical stability for LMNPs due to surface passivation. Thus, polymers bearing multidentate phosphonic acids are desirable to decorate LMNPs to meet complex environments for biological studies.

## Introduction

In the past decade, gallium (Ga)-based liquid metal (LM) alloys (e.g., eutectic gallium–indium, EGaIn; and gallium–indium–tin, Galinstan), as metallic fluids at room temperature, have emerged as new generation soft functional materials due to their unique combination of fluidic flexibility, standard bulk metallic properties, surface reactivity, and biocompatibility.^1−3^ Many of their unique characteristics are desirable in cutting-edge biomedical applications. For instance, the high electrical conductivity of LMs makes them responsive to electric and magnetic fields, bestowing them with the ability to be controlled remotely;^4−6^ the low viscosity and high fluidity allow LMs to be shape transformable in response to stress, making them naturally conformable to soft biological tissues;^7−9^ the reactive surface of Ga (and its oxide) renders LMs with surface functionalization accessibility, which also enables the morphological transformation caused by chemical reactions;^10−12^ plus, the combination of fluidity and surface reactivity allows LMs to be broken up readily to form micro-/nanosized particles via various top-down approaches.^7,13−16^ Despite the above-mentioned desirable features of Ga-based LMs, there are several challenges preventing their practical biorelated applications, of which the major weakness lies in controllable physical and chemical properties (e.g., controlled particle size and surface properties) that should be diverse to meet the complex requirement for translation in biomedical fields.^15,17^ In recent years, the development of LM nanocomposites in synergy with functional polymers has shown great potential in tackling this challenging issue by tuning the intrinsic properties of LMs for desired bioapplications.^7,18^

Currently, commonly used strategies to obtain polymer/LM nanocomposites involve the direct preparation of LM nanoparticles (LMNPs) in an aqueous solution of polymers through mechanical agitation, microfluidics, or ultrasonication methods.^13,19−22^ Surface grafting through the anchoring groups, such as thiols,^23−25^ catechols,^21,26^ phosphonic acids,^27,28^ trithiocarbonates,^13,19^ carboxylic acids,^29,30^ silanes,^31^ and amines,^12,32^ is generally required to prevent the detachment of polymers. However, due to the low reaction temperature required for liquid metals, the coordination between polymers and metals is weaker than that of the particles prepared through high-temperature approaches.^33^ Additionally, the presence of a great excess of water strongly influences the surface coating of stabilizing polymers, which can be mainly attributed to the strong affinity of water to metal ions.^34,35^ Most importantly, the biological application of polymers/LMNPs often requires satisfactory colloidal stability in biological buffers and cell culture media (CCM).^15^ The high ion concentration of these isotonic buffered salt solutions will lead to the compression of the electric double layer to further induce the collapse of LMNPs, thereby inducing the formation of aggregates. Given that multiple surface coating strategies have been reported for the stabilization of LMNPs, there is still a lack of information on the colloidal stability in different biological solutions. Indeed, relatively little research has been done to systemically investigate how the binding hierarchy of different ligands affects the colloidal stability of LMNPs in biological solutions and how to engineer the grafting polymer to optimize both the colloidal and chemical stabilities of LMNPs. Consequently, there are limited polymers available for the preparation of highly stable LMNPs for biomedical applications.

**Figure 1:**
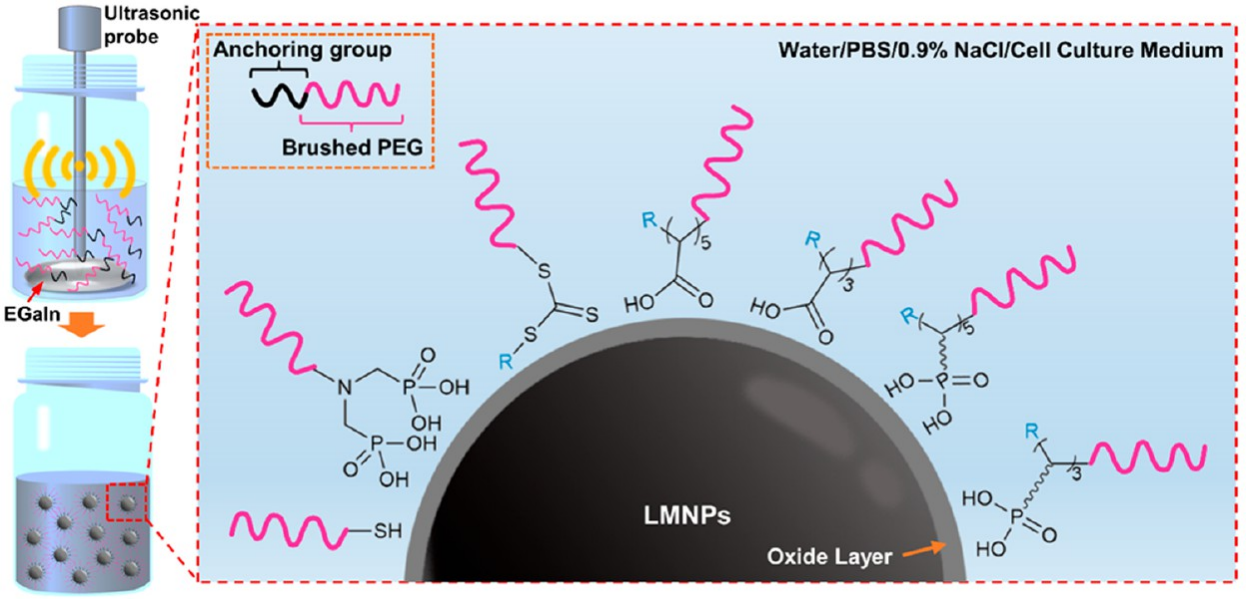
Production of polymer grafted LMNPs in polymer solutions via ultrasonication. The colloidal and chemical stabilities of LMNPs grafted by bPEG polymers bearing a variety of anchoring groups were systematically investigated in biological buffers.

Here, we report the engineering of polymers for the surface grafting of Ga-based LMNPs toward high stability in water, physiological buffer solution, and CCM. Different anchoring groups were incorporated into polymers through RAFT polymerization. The colloidal stability of as-prepared LMNPs was systemically investigated to evaluate the binding hierarchy of different anchoring groups. Thereafter, polymers with multidentate ligands were designed and synthesized to improve the surface binding affinity to LMNPs and the number of anchoring groups was evaluated and determined based on their stability in biological buffers (Figure 1).

## Results and Discussion

### Design and Synthesis of Brushed Polymers Bearing Different Anchoring Groups

To study the surface anchoring effect, we first designed a series of brushed polymers (bPEG) with different anchoring moieties, including trithiolcarbonate (TTC), thiol (HS), and diphosphonic acid (DiPA) groups (Figure S1), as these groups have been previously reported with relatively high binding affinities to Ga-based LMs.^13,15,27^ The polymers were prepared through RAFT polymerization by using a chain transfer agent (CTA) terminated with diphosphonate (diphosphonate-CTA) owing to the presence of protected DiPA and TTC group and facile postmodification to expose the thiol group.^36^^1^H NMR and ^31^P NMR spectra as shown in Figure S2 indicate the successful synthesis of diphosphonate-CTA, in which the chemical shift of the phosphorus signals is 26.62 ppm. Hydrophilic TTC-terminated PEG brush polymers were simply obtained by RAFT polymerization of oligo(ethylene glycol) methyl ether acrylate (OEGA, *M*_n_ = 480) and denoted as TTC-bPEG (Figure S3). Followed by deprotection of the diphosphonate group, DiPA-terminated PEG brush polymers (DiPA-bPEG) were also obtained and confirmed by ^1^H NMR spectroscopy as shown in Figure S4. HS-terminated PEG brush polymers (HS-bPEG) were prepared through aminolysis of TTC-bPEG with excess amounts of butylamine (Figure S5).^37^

Beyond different anchoring groups, we also designed a series of bPEG with different numbers of anchoring groups to clarify the influence of multidentate ligands on the colloidal, morphological, and chemical stabilities of LMNPs. Recently, the carboxylic acid (CA) was reported as a strong anchoring group that could attach onto the Ga oxide “skin” layer via multiple coordination interactions.^30^ Besides, phosphonic acid (PA) possibly exhibits higher binding affinity to the Ga oxide due to the formation of bidentate or tridentate gallium oxide–PA bonds.^27,28^ Thus, 2-(((butylthio)carbonothioyl)thio)propanoic acid (BTPA) was synthesized as CTA to incorporate CA or PA into the PEG brush polymers (Figure S6). PA monomer was simply synthesized through an 1-Ethyl-3-(3-dimethylaminopropyl)carbodiimide (EDC) coupling reaction between acrylic acid and dimethyl (2-hydroxyethyl)phosphonate and verified by ^1^H NMR (Figure S7). BTPA-PEG brush polymers were prepared as macro-CTA for the second polymerization to incorporate different numbers of CA or PA groups (Figure S8). In the second polymerization, acrylic acids and PA monomers were incorporated with different numbers. The CA- or PA-terminated bPEG was confirmed by ^1^H NMR (Figures S9 and S10) and denoted as CA_3_-bPEG, CA_5_-bPEG, PA_3_-bPEG, and PA_5_-bPEG, respectively, in which the number of CA or PA groups was calculated according to the feed ratios and conversion rates of the monomers. Thus, we obtain a series of polymers with similar molecular weights for the fabrication of polymers/LMNPs as summarized in Table 1 and Figure S11.

**Table 1:**
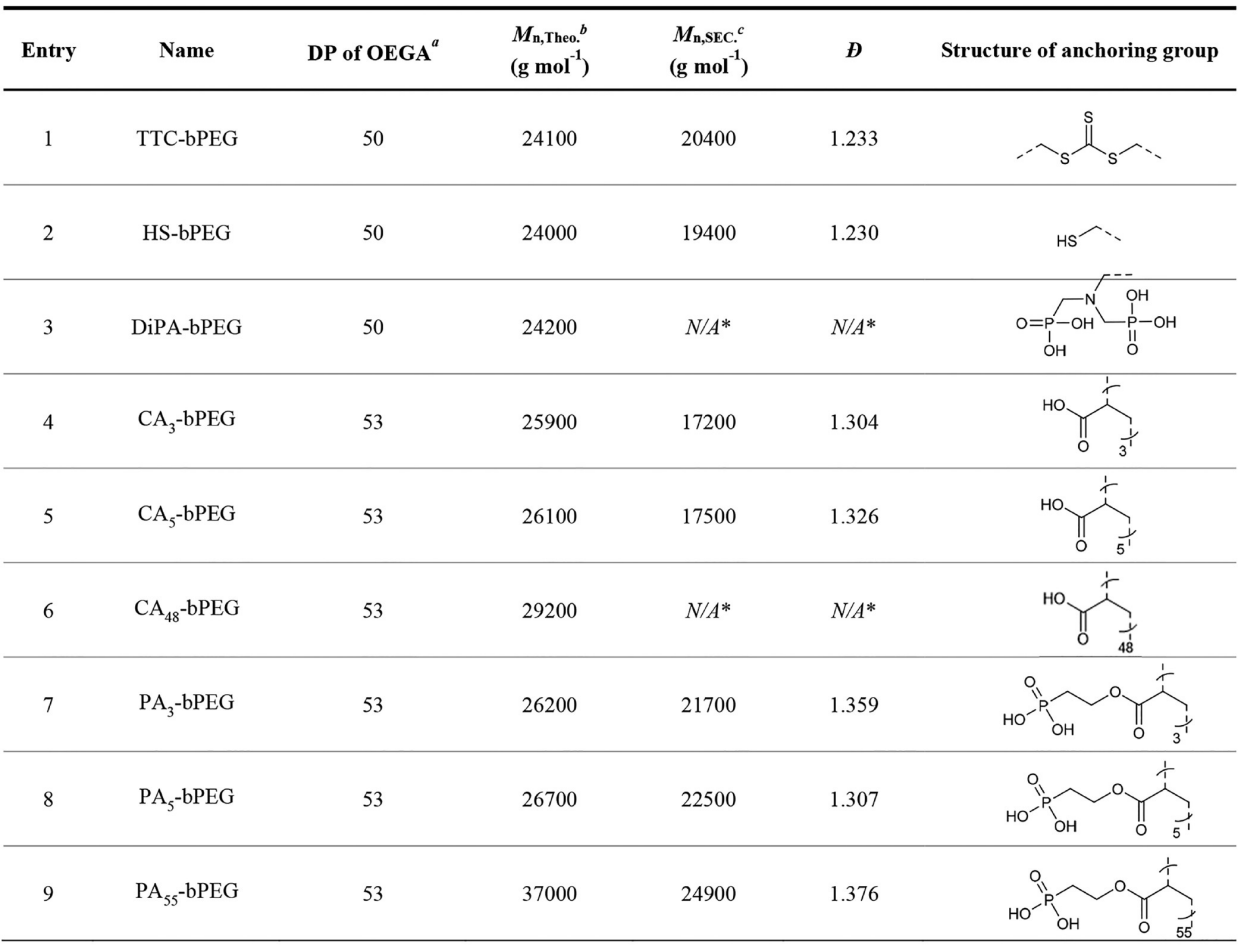
PEG Brush Polymers Bearing Different Anchoring Groups Synthesized via RAFT Polymerization

All polymerizations were conducted in DMF at 70 °C using AIBN as an initiator; the reaction times are 4 h for the first step polymerization and 12 h for the second polymerization.

a Degree of polymerization was calculated from the conversion rate and feed ratio.

b Theoretical molecular weight was calculated from the conversion rate (*M*_n,Theo._ = number of monomer_equiv. to CTA_ × conversion rate × *M*_monomer_ + *M*_CTA_).

c Number-average molecular weight was determined by DMF SEC using PS as standard.

* SEC result of DiPA-bPEG and CA_48_-bPEG was not obtained due to the strong interaction to the SEC column.

### Evaluation of Colloidal and Chemical Stabilities of Polymers/LMNPs with Various Anchoring Groups

Till now, it has still been challenging to stabilize LMNPs within aqueous media despite the fact that various anchoring groups were used to stabilize LMNPs (e.g., thiol, catechol, phosphonic acid, trithiocarbonate, carboxylic acid, amine, etc.). TTC-, HS-, and DiPA-terminated hydrophilic polymers were chosen in our work for their facile preparation using RAFT CTA. In general, the bPEG polymers bearing different anchoring groups and one droplet of bulk EGaIn were first dissolved in Milli-Q water and precooled under an ice bath to avoid overheating of liquid during the sonication. Then, the solution was sonicated for 40 min under an ice bath (illustrated in Figure 1). The bulk materials and aggregates were removed by centrifugation at 1,000 rpm for 10 min, obtaining the supernatant as polymers/LMNPs as summarized in Table S1. The successful grafting of polymers to LMNPs was confirmed by FT-IR spectra, in which the C–H stretch, C=O stretch, C–H bend, and C–O stretch signals on polymers and polymers/LMNPs are consistent as shown in Figures S12 and S13.

With the increasing attempts to apply LMNPs toward biomedical applications, understanding the colloidal stabilities of LMNPs in different biological environments is critical for the further clinical translation of LMNP-based nanomaterials. Herein, we chose four different aqueous solutions to demonstrate the colloidal stabilities and chemical stabilities of LMNPs, including Milli-Q water, phosphate buffer saline (PBS), 0.9% sodium chloride (0.9% NaCl) solution, and 10% FBS Dulbecco’s Modified Eagle Medium (DMEM) culture medium. Water and PBS solutions are the most commonly used aqueous solutions in biological research, in which the osmolarity and ion concentrations of PBS match those of the human body. Besides, 0.9% NaCl injection is commonly used for replacement of the lost body fluids and salts and administration of medicines via injection. Despite the fact that the culture medium of cells depends on the type of cells, 10% FBS DMEM presents one of the most common cell culture media, providing the nutrients for cell survival, growth, and differentiation.^38^ In our study, polymers/LMNPs were diluted to 2 mg/mL by different aqueous solutions, and all the subsequent experiments were conducted at room temperature (∼23 °C). Despite the fact that an increasing number of researchers recently reported numerous biomedical applications of LMNPs, such as cancer treatment,^32,39−41^ medical imaging,^32,42^ ion channel regulation,^32^ and pathogen treatment,^43,44^ the majority of studies on examining the stability of LMNPs were conducted only in water or organic solutions without complex ionic environments. To systematically investigate the stability, PBS, 0.9% NaCl solution, and 10% FBS DMEM culture medium were chosen to mimic the applied environment of LMNPs toward bioapplications. The colloidal stabilities of bare LMNPs and LMNPs grafted with bPEG bearing TTC, HS, and DiPA groups (denoted as TTC-bPEG/LMNPs, HS-bPEG/LMNPs, and DiPA-bPEG/LMNPs) were monitored by DLS and photographs up to 7 days.

**Figure 2:**
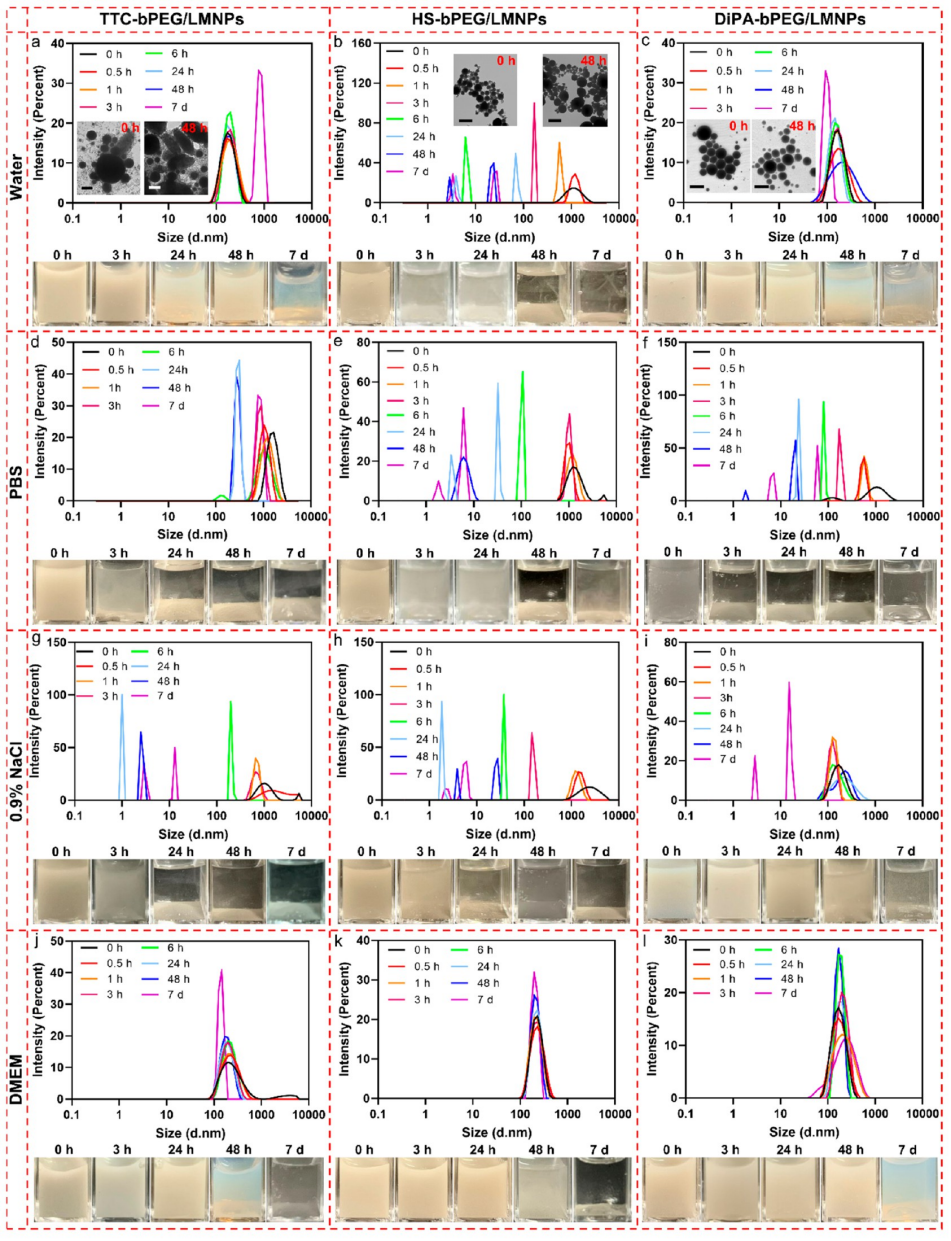
Investigation of colloidal stability for TTC-bPEG/LMNPs, HS-bPEG/LMNPs, and DiPA-bPEG/LMNPs. Hydrodynamic size distribution and photographs of TTC-bPEG/LMNPs, HS-bPEG/LMNPs, and DiPA-bPEG/LMNPs in water (a–c), PBS (d–f), 0.9% NaCl (g–i), and 10% FBS DMEM culture medium (j–l) at different time points. Insets of parts a–c show TEM images of LMNPs at 0 and 48 h. Scale bars are 100 nm.

Bare LMNPs without polymer coating exhibit poor stability in all aqueous solutions, as shown in Figure S14. In water, small particles can be suspended for 48 h due to the heterogenicity of the particle size, which can also be verified by transmission electron microscopy (TEM) images (see insets of Figure S14a). In PBS and 0.9% NaCl, all bare LMNPs precipitated within 3 h (Figures S14b–d). The chemical stability of bare LMNPs was also unstable, in that the LMNPs were totally oxidized and hydrolyzed to form oxide nanorods in 48 h (Figure S14e). In water, TTC-bPEG/LMNPs had a hydrodynamic size peak at 160 nm and they showed good colloidal stability up to 48 h after production, which was also evidenced by the photographs taken for TTC-bPEG/LMNP suspension (Figure 2a). We observed a significant increase in hydrodynamic size and sedimentation of nanoparticles 7 days after production. The insets of Figure 2a showed an increased number of nanorods as time passes (will discuss this later). On the contrary, HS-bPEG/LMNPs showed very poor colloidal stability even just right after preparation, as evidenced by the large DLS size distribution peak (∼1200 nm at 0 h) and photographs obtained for the suspension (Figure 2b). Aggregation and sedimentation occurred within 30 min, and the supernatant became clear and almost fully transparent at 48 h, in which the hydrodynamic size decreased to lower than 10 nm corresponding to the size of free hydrophilic HS-bPEG. However, no nanorods were formed 48 h after production (see Figure 2b insets). As for DiPA-bPEG/LMNPs, they presented relatively good colloidal stability in water until 48 h (Figure 2c), after which the suspension became more transparent than that of TTC-bPEG/LMNPs. Sedimentation was also observed for DiPA-bPEG/LMNPs after 24 h storage at room temperature. Again, no nanorods were formed 48 h after production (see Figure 2c insets). Unlike the cases of water, all three polymers/LMNPs systems exhibited poor colloidal stability in PBS (Figure 2d–f), with all of them showing a large DLS distribution peak at around 1,000 nm even right after production. More specially, DiPA-bPEG/LMNPs exhibited the poorest stability, in which the supernatant became transparent at 3 h. High-ionic-strength 0.9% NaCl solution was also used to evaluate the stability of these three polymer/LMNP systems. In 0.9% NaCl solutions, TTC-bPEG/LMNPs and HS-bPEG/LMNPs both rapidly sedimented within 24 h (Figure 2g and h). However, DiPA-bPEG/LMNPs still exhibit relatively good stability until 48 h, as evidenced by the broadened DLS size distribution (Figure 2i). Comparing the results of DiPA-bPEG/LMNPs in 0.9% NaCl and PBS, the poor stability of DiPA-bPEG/LMNPs in PBS may be attributed to the competitive binding between DiPA-bPEG and phosphate saline. Such competitive association and disassociation are common in polymer-stabilized nanoparticle systems.^45^

When dispersed in 10% FBS DMEM, all these three polymer/LMNP systems showed similar stability behaviors compared to that in water. Among them, TTC-bPEG/LMNPs and HS-bPEG/LMNPs exhibit slightly worse colloidal stability (Figure 2j and k). On the other hand, DiPA-bPEG/LMNPs are more stable in DMEM, in which the degree of sedimentation is lower at 48 h and 7 days compared to that in water (Figure 2l). This set of experiments indicates that the DiPA group exhibits the highest binding affinity to LMNPs in CCM such as 10% FBS DMEM. DMEM contains 0.92 mM phosphate ions, which is approximately 10 times less than that of PBS, mitigating the competitive association phenomenon. Notably, the total protein content in DMEM culture medium is near 3.0–4.5 g L^–1^ containing 10.65 mM amino acids,^46^ which could also act as anchoring groups to the LMNPs and play an important role in improving the stability.

**Figure 3:**
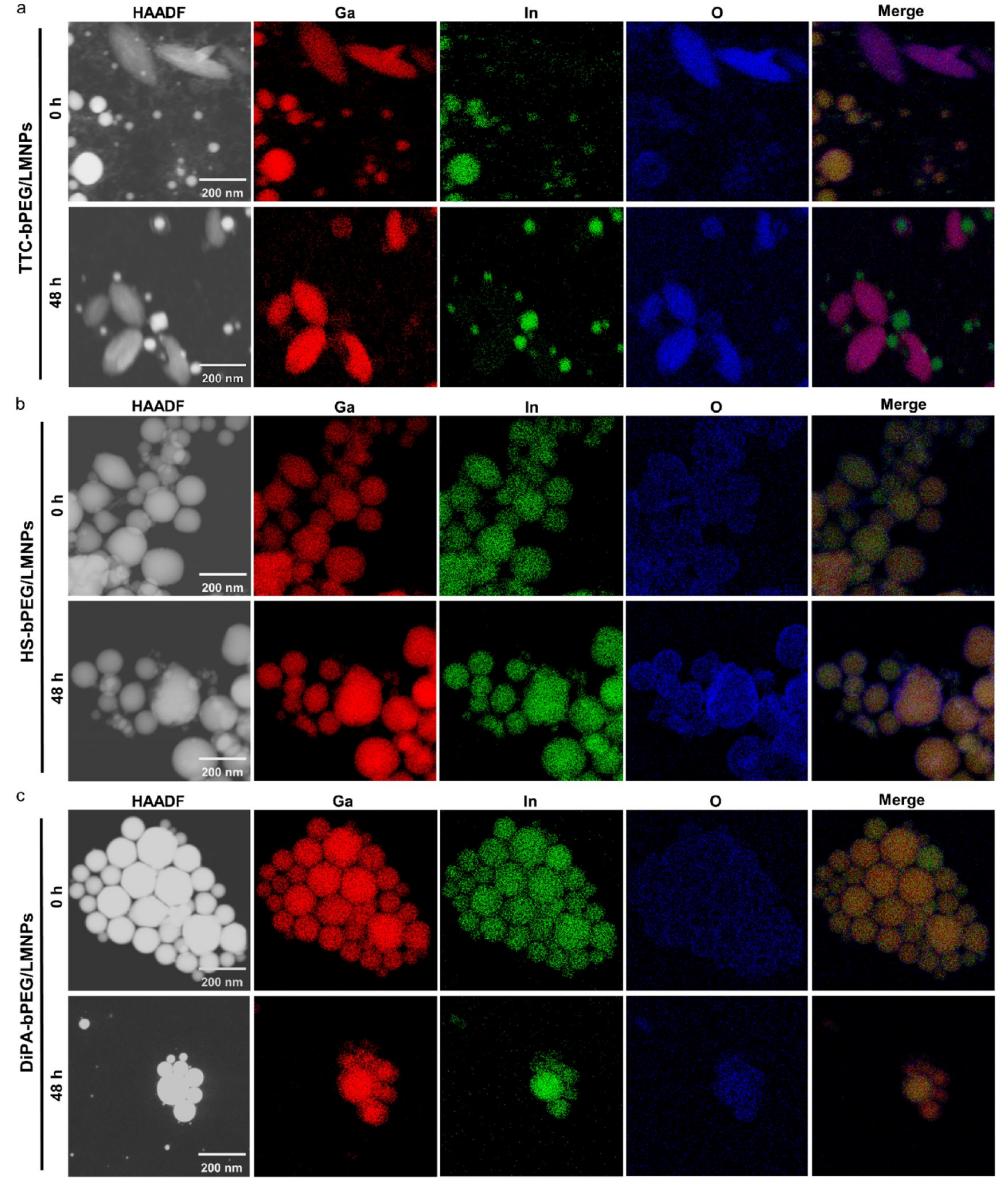
Morphological and chemical stability investigation of TTC-bPEG/LMNPs, HS-bPEG/LMNPs, and DiPA-bPEG/LMNPs in water. EDS mapping of TTC-bPEG/LMNPs (a), HS-bPEG/LMNPs (b), and DiPA-bPEG/LMNPs (c) at 0 and 48 h.

We further evaluated the chemical stabilities of LMNPs grafted with bPEG bearing different anchoring groups. The shape transformation (oxidation and dealloying) mechanism of LMNPs has been systematically investigated,^10,12^ in which Ga was oxidized to Ga oxide (Ga_2_O_3_) and then gradually hydrolyzed by H_2_O to form the Ga oxide monohydroxide (GaOOH). Transmission electron microscopy (TEM) and energy-dispersive X-ray spectroscopy (EDS) mapping were conducted to reveal the morphologies and elemental constitutions of TTC-bPEG/LMNPs, HS-bPEG/LMNPs, and DiPA-bPEG/LMNPs suspended in water 0 and 48 h after production (Figure 3). The high-angle annular dark-field (HAADF) image of TTC-bPEG/LMNPs at 0 h given in Figure 3a reflects the coexistence of spherical nanoparticles and rice-like nanorods (see also TEM images given in Figure 2a insets), in which the rice-like nanorods present the early stage of dealloying of TTC-bPEG/LMNPs.^47^ The EDS mappings at 0 h confirm overlap of Ga, indium (In), and O elements within the spherical LMNPs, corresponding to the liquid EGaIn nanoparticles (Figure 3a). The rice-like TTC-bPEG/LMNPs were composed of Ga and O elements, indicating the formation of GaOOH but still at the early stage of dealloying of TTC-bPEG/LMNPs.^10^ After 48 h, the morphology of TTC-bPEG/LMNPs was predominantly rice-like nanorods and only a small number of spherical nanoparticles were observed. Notably, the HAADF image for TTC-bPEG/LMNPs shows a few unique spherical particles, in which a thick shell encapsulates the bright white core. The EDS mapping indicated that the elemental constitutions of the thick shell are Ga and O corresponding to the Ga_2_O_3_, while the inner core is In rich (Figure 3a). All these results demonstrate that TTC is a poor ligand to maintain the chemical stability of LMNPs.

The morphologies and elemental constitutions of HS-bPEG/LMNPs and DiPA-bPEG/LMNPs were also characterized in water 0 and 48 h after production, as shown in Figure 3b and c, respectively. HS-bPEG/LMNPs at 0 and 48 h were spherical nanoparticles with slight roughness on the surface, demonstrating mild oxidation of Ga. The slightly severe oxidation was observed at 48 h compared to HS-bPEG/LMNPs at 0 h (Figure 3b). The EDS mapping results of DiPA-bPEG/LMNPs demonstrate that the morphology and elemental constitutions were highly preserved in 48 h without significant oxidation (Figure 3c), indicating the surface passivation functionality of PA similar to our previously reported results.^28^

After systematic evaluation of the colloidal and chemical stabilities of LMNPs grafted with TTC-, HS-, and DiPA-terminated bPEG, we demonstrate that the DiPA group exhibits the highest binding affinity to the Ga oxide surface, yielding high colloidal stability, and meanwhile protects the LMNPs from further oxidation to form GaOOH nanorods. The TTC group exhibited moderate binding affinity to the Ga oxide surface to achieve moderate colloidal stability in all four types of aqueous solutions; however, it does not protect the LMNPs from oxidation, forming rice-like GaOOH nanorods. HS-bPEG/LMNPs showed the poorest colloidal stability among the three anchoring groups, but the spherical shape of LMNPs was preserved, which may be attributed to the rapid sedimentation limiting the accessibility of oxygen and H_2_O.

### Influence of Multidentate Ligands on the Colloidal and Chemical Stabilities of Polymers/LMNPs

**Figure 4:**
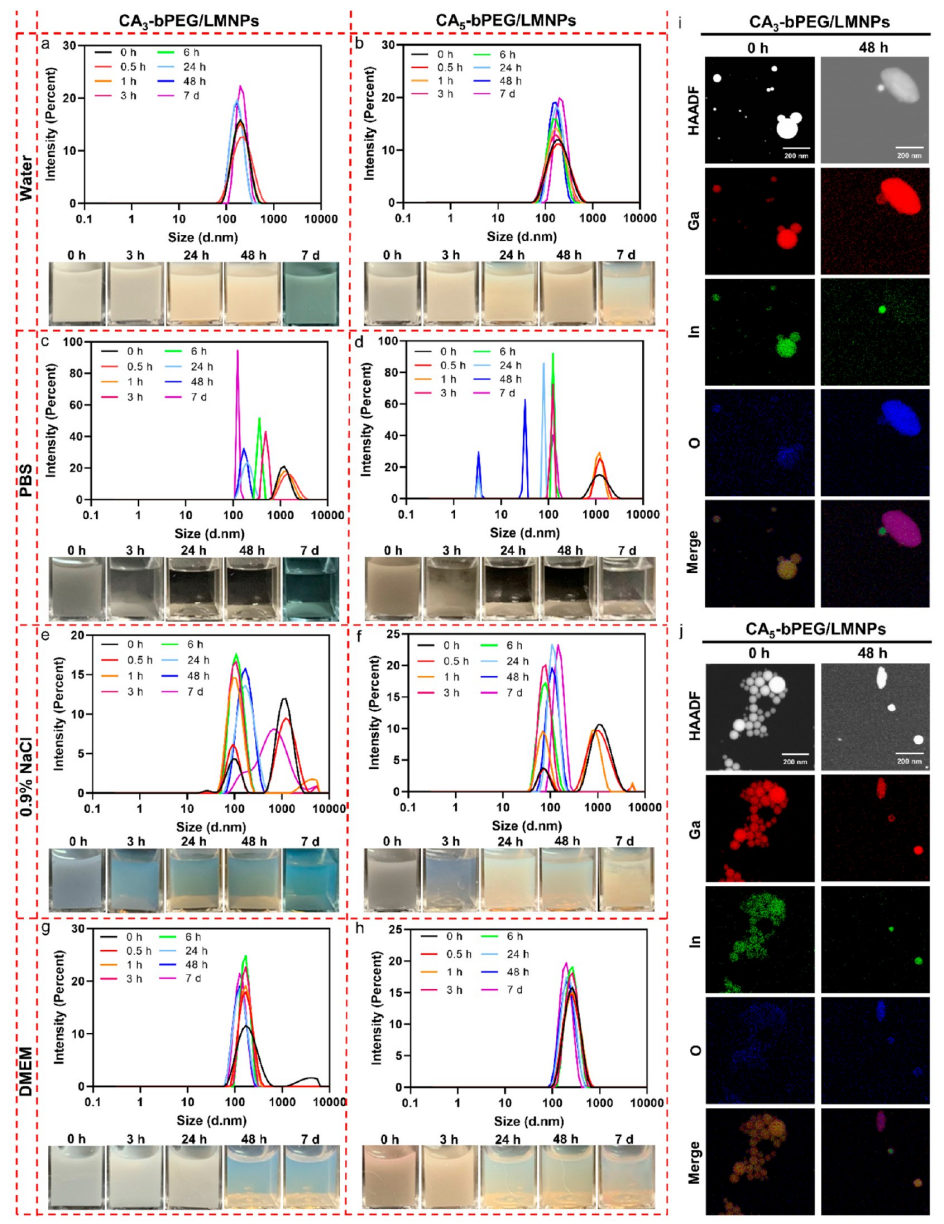
Evaluation of colloidal and chemical stabilities of CA_3_-bPEG/LMNPs and CA_5_-bPEG/LMNPs. Hydrodynamic size distributions and photographs of CA_3_-bPEG/LMNPs and CA_5_-bPEG/LMNPs in water (a and b), PBS (c and d), 0.9% NaCl (e and f), and 10% FBS DMEM culture medium (g and h) at different time points. EDS mappings of CA_3_-bPEG/LMNPs (i) and CA_5_-bPEG/LMNPs (j) 0 and 48 h after production in water.

Based on the above-obtained results, we hypothesize that multidentate ligands may further enhance the colloidal and chemical stability of LMNPs. To study this, we designed a series of bPEG in which different numbers of CA and PA groups were incorporated via RAFT polymerization. The DLS size distributions and photographs for CA_*x*_-bPEG grafted LMNPs in different biological media are summarized in Figure 4. Both CA_3_-bPEG/LMNPs and CA_5_-bPEG/LMNPs exhibited excellent colloidal stability in water for 7 days, with a narrow size distribution peak at around 150 nm. Despite the slight sedimentation at 7 days, the colloidal stability is still better than that of TTC, HS, and DiPA groups, indicating that multidentate ligands benefit the colloidal stability of LMNPs. There is no significant difference in colloidal stability when increasing the number of CA groups, although the DLS size distribution became more uniform when using bPEG with more CA groups. In PBS solution, the colloidal stability is poor for both CA_3_-bPEG/LMNPs and CA_5_-bPEG/LMNPs, in which the sedimentation occurred at 3 h and the supernatants became transparent at 24 h (Figure 4c and d). PBS is the most severe environment among the four solutions for stabilizing LMNPs. The colloidal stabilities of CA_3_-bPEG/LMNPs and CA_5_-bPEG/LMNPs in 0.9% NaCl were moderate, with sedimentation observed 3 h after production (Figure 4e and f). In the 10% FBS DMEM culture medium, the colloidal stability is better than those in 0.9% NaCl and PBS groups but worse than that in water (Figure 4g and h). However, we found that CA groups failed to chemically stabilize LMNPs, with the formation of rice-shaped GaOOH observed within 48 h after production (Figure 4i and j; see also Figure S15 for TEM images).

**Figure 5:**
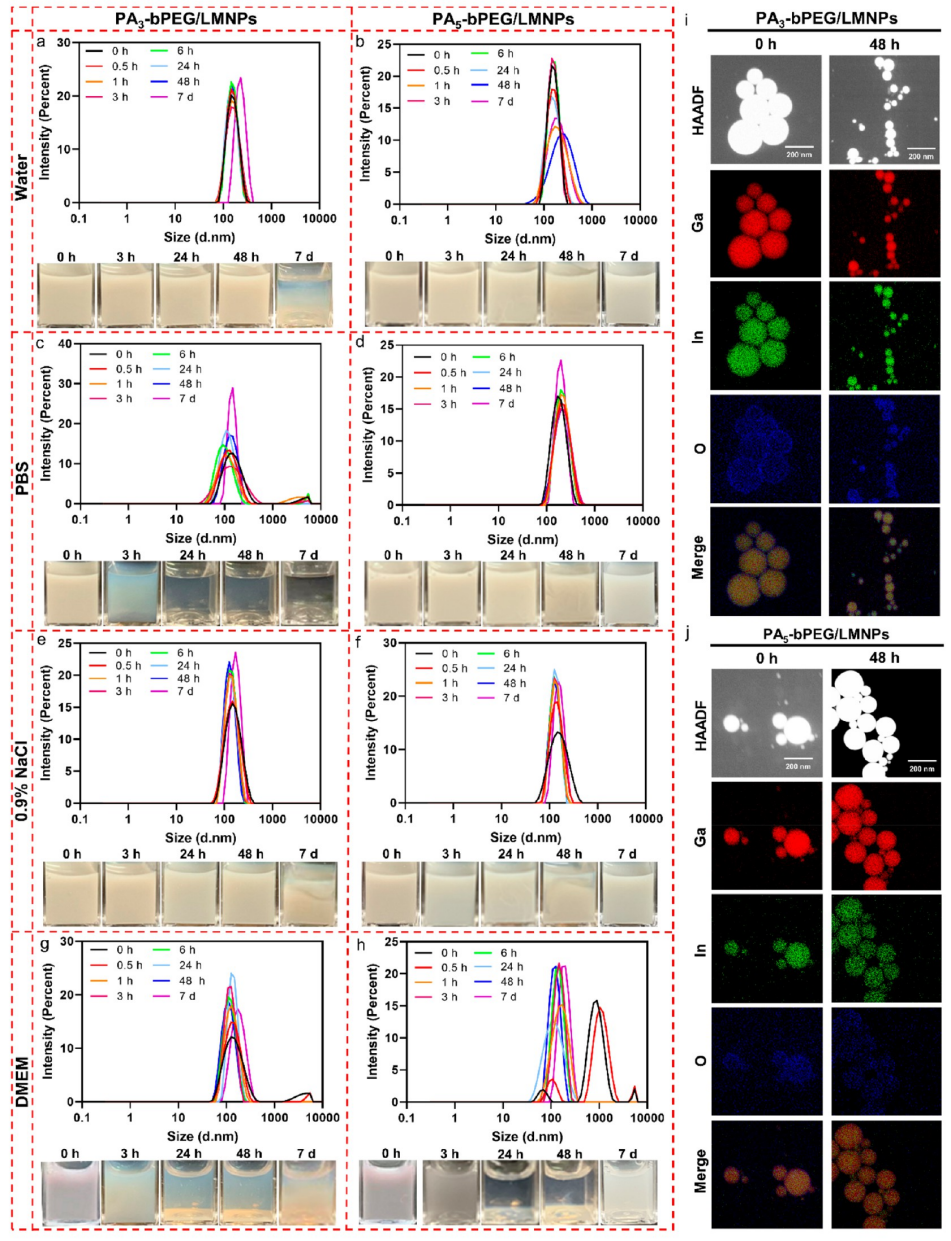
Evaluation of colloidal and chemical stabilities of PA_3_-bPEG/LMNPs and PA_5_-bPEG/LMNPs. Hydrodynamic size distributions and photographs of PA_3_-bPEG/LMNPs and PA_5_-bPEG/LMNPs in water (a and b), PBS (c and d), 0.9% NaCl (e and f), and 10% FBS DMEM culture medium (g and h) at different time points. EDS mappings of PA_3_-bPEG/LMNPs (i) and PA_5_-bPEG/LMNPs (j) 0 and 48 h after production in water.

As for LMNPs grafted with bPEG bearing three or five PA groups (denoted as PA_3_-bPEG/LMNPs and PA_5_-bPEG/LMNPs, respectively), we conducted similar stability characterizations in water, PBS, 0.9% NaCl, and 10% FBS DMEM culture medium. Both PA_3_-bPEG/LMNPs and PA_5_-bPEG/LMNPs exhibit excellent colloidal stability within 48 h, showing a narrow DLS size distribution peak at around 150 nm in water (Figure 5a and b). PA_5_-bPEG/LMNPs remained stable even 7 days after production (DLS peak of 169 nm, PDI = 0.155), which may be attributed to the preponderant binding affinity of PA_5_-bPEG. However, the poor stability of PA_3_-bPEG/LMNPs in PBS is also observed, in which LMNPs fully sedimented in 24 h (Figure 5c). Interestingly, increasing the number of PA to five provides PA_5_-bPEG/LMNPs with excellent colloidal stability in PBS, showing no obvious sedimentation in 7 days (Figure 5d). PA_3_-bPEG/LMNPs and PA_5_-bPEG//LMNPs also have similar excellent stabilities in 0.9% NaCl solution (Figure 5e and f); however, their stabilities became much worse in the 10% FBS DMEM culture medium (Figure 5g and h). The reason behind the compromised stability in DEME is still subject to future investigation. In addition, similar to the case of DiPA-bPEG/LMNPs where PA groups can passivate LMNPs, PA_3_-bPEG/LMNPs and PA_5_-bPEG/LMNPs remained chemically stable in water for at least 48 h, showing no formation of GaOOH nanorods (Figure 5i and j; see also Figure S16 for TEM images).

**Figure 6:**
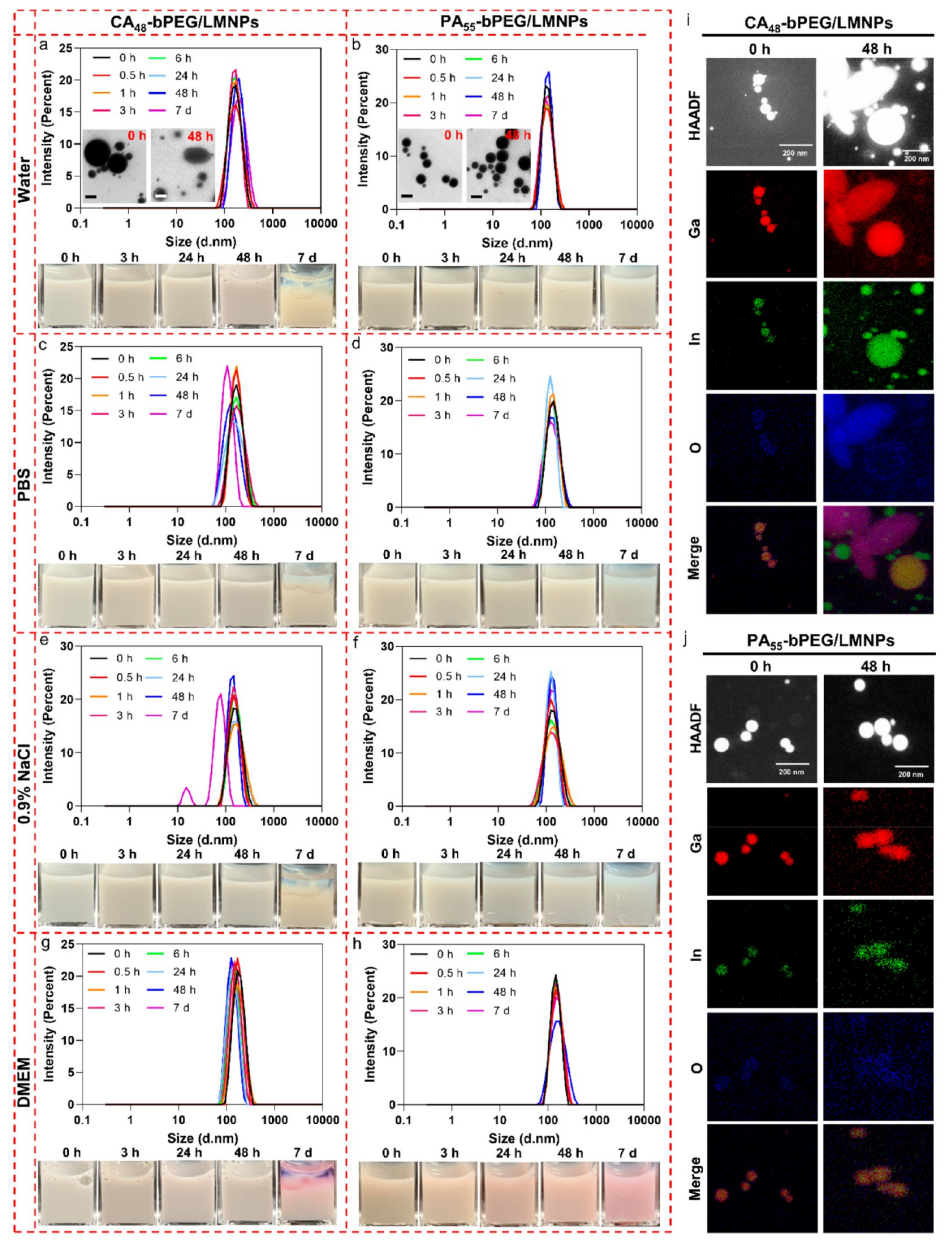
Evaluation of colloidal and chemical stabilities of CA_48_-bPEG/LMNPs and PA_55_-bPEG/LMNPs. Hydrodynamic size distributions and photographs of CA_48_-bPEG/LMNPs and PA_55_-bPEG/LMNPs in water (a and b), PBS (c and d), 0.9% NaCl (e and f), and 10% FBS DMEM culture medium (g and h) at different time points. EDS mappings of CA_48_-bPEG/LMNPs (i) and PA_55_-bPEG/LMNPs (j) 0 and 48 h after production in water.

Apart from three or five ligands, we further increased the number of anchoring groups up to around 50. CA_48_-bPEG/LMNPs exhibit outstanding colloidal stability within 48 h in all four aqueous solutions due to the significant increase of the numbers of anchoring groups. When compared with CA_5_-bPEG/LMNPs and CA_3_-bPEG/LMNPs, the size distribution of CA_48_-bPEG/LMNPs was more uniform and the sedimentation was unconspicuous even in 7 days (Figure 6a), further indicating the influence of multidentate ligands on the colloidal stability of LMNPs. However, even 48 CA groups still cannot protect the LMNPs from shape transformation after 48 h storage at room temperature, as shown in Figure 6a and i. As for PA_55_-bPEG/LMNPs, they possessed excellent colloidal and chemical stabilities even up to 7 days, verified by DLS, photographs, TEM images, and EDS mapping as shown in Figure 6.

## Conclusion

In this work, for the first time, we systematically investigated the influence of anchoring groups on the colloidal and chemical stabilities of LMNPs. TTC-, HS-, DiPA-, CA_3_-, CA_5_-, CA_48_-, PA_3_-, PA_5_-, and PA_55_-terminated bPEG polymers were synthesized via RAFT polymerization to decorate the EGaIn nanoparticles. LMNPs grafted with different anchoring groups were first investigated, exploiting DLS, photographs, TEM, and EDS mapping. In brief, compared to TTC and HS, DiPA groups exhibit the highest colloidal stability and can prevent the shape transformation of LMNPs in water at room temperature. The poor colloidal stabilities of DiPA-bPEG/LMNPs observed in PBS can be attributed to the competitive binding between DiPA and phosphate ions. Our results also indicate that LMNPs grafted with non-PA anchoring groups can induce spontaneous shape transformation in water (caused by oxidation and hydrolysis) at room temperature. Moreover, we also choose CA and PA groups to investigate the influence of multidentate ligands on the colloidal and chemical stabilities of LMNPs. Three, five, or fifty CA or PA groups were incorporated via a second RAFT polymerization process. According to our results, more anchoring groups can lead to better colloidal stability, where PA_55_-bPEG/LMNPs exhibit the best stability in water, PBS, and 0.9% NaCl up to 7 days. However, increasing the number of anchoring groups will inevitably increase the cost of polymer preparation and it is difficult to predict the influence of excess anchoring groups when introducing them into biological environments. It is possible that excess anchoring groups will interact with proteins or other molecules in biological environments, which needs further investigation in related biological applications. To conclude, this study may pave the way for the development of future stable biomedical platforms based on polymer/LMNP systems.

## Experimental Section

### Materials

All the chemicals and solvents were purchased from Sigma-Aldrich or Merck and used as received unless otherwise stated. Dulbecco’s Modified Eagle Medium (DMEM) culture medium, penicillin-streptomycin-glutamine (100×), and fetal bovine serum (FBS) were purchased from Gibco (Grand Island, NY, USA) to prepare 10% FBS DMEM culture medium.

### Synthesis of 2-(((Butylthio)carbonothioyl)thio)propanoic Acid (BTPA) as Chain Transfer Agent (CTA)

BTPA as CTA was synthesized according to the previous report.^48^ Typically, 1.78 g (44.5 mmol) of NaOH was dissolved in 25 mL of Milli-Q water in a round-bottom flask under ice bath and 4 g (44.5 mmol) of 1-butanethiol (99%) was added dropwise to the NaOH solution under stirring. After reacting at room temperature for 30 min, 3.38 g (44.5 mmol) of carbon disulfide (ACS reagent, ≥99.9%) was added into the reaction solution. The solution turned yellow immediately and reacted for another 4 h. 6.8 g (44.5 mmol) of 2-bromopropanoic acid (99%) dissolved in 10 mL of Milli-Q water was slowly added into the 15 mL NaOH solution containing 1.78 g (44.5 mmol) of NaOH. The 2-bromopropanoic acid solution was then added into the reaction solution in one portion and reacted overnight. One equivalent of HCl solution was slowly added into the reaction solution, and crude products were obtained through extraction using ethyl acetate (3 × 50 mL). The products were purified by FLASH column chromatography (10% to 20% ethyl acetate/hexane) and evaporated to obtain the final products as a yellow solid.

### Synthesis of Diphosphonate-CTA

Synthesis of diphosphonate-CTA has been previously reported by our group.^36^ Tetramethyl(((2-hydroxyethyl)azanediyl)bis(methylene))bis(phosphonate) (0.610 g, 2 mmol) was dissolved in 3 mL of anhydrous dichloromethane (DCM) with 300 μL (0.202 g, 2 mmol) of triethylamine and degassed with N_2_ for 15 min under ice bath. Then 2-bromopropanoyl bromide (0.428 g, 2 mmol) was added dropwisely, and then the mixture was reacted overnight. The solution was filtered and washed with saturated NaHCO_3_ 3 times and H_2_O 3 times and dried with MgSO_4_. After evaporation of solvent, 2-(bis((dimethoxyphosphoryl)methyl)amino)ethyl 2-bromopropanoate was obtained.

1-Butanethiol (0.307 g, 3.4 mmol) was dissolved in 10 mL of anhydrous DCM in a 100 mL flask. Then 1.5 mL of triethylamine and then carbon disulfide (0.259 g, 3.4 mmol) were added dropwisely, and the mixture was stirred at room temperature for 2 h. Next, 2-(bis((dimethoxyphosphoryl)methyl)amino)ethyl 2-bromopropanoate (4.39 g, 0.01 mol) in 3 mL of DCM was added dropwisely, and the mixture was stirred for overnight at room temperature. The reaction mixture was extracted with water 3 times, dried with MgSO_4_, and evaporated to obtain crude RAFT CTA. FLASH column chromatography (*n*-hexane/ethyl acetate = 1:1) was used to purify the product.

### Synthesis of 2-(Dimethoxyphosphoryl)ethyl Acrylate

Dimethyl (2-hydroxyethyl)phosphonate (2.00 g, 12.98 mmol) and acrylic acid (1.12 g, 15.57 mmol) were mixed in 20 mL of tetrahydrofuran (THF) under ice bath. DMAP (0.05 equiv) was added to the reaction mixture as catalyst. EDC (2.42 g, 15.57 mmol) dissolved in 20 mL of THF was added dropwise, and the reaction was left at room temperature for 48 h after complete addition. The crude mixture was obtained after washing with water (3 × 20 mL) and drying over anhydrous MgSO_4_, followed by evaporation of excess solvent. Then, the crude mixture was purified by FLASH column chromatography (10% to 40% DCM/THF) to obtain the target compound as a colorless liquid.

### Synthesis of bPEG Polymer

RAFT polymerization was conducted in a typical procedure: 100 mg (0.19 mmol) of diphosphonate-CTA, 3.12 mg (0.02 mmol) of AIBN, and 5023.18 mg (10.46 mmol) of EGA monomer were mixed in 5 mL of dimethylformamide (DMF) in a 25 mL round-bottom flask. The reaction mixture was sealed with septa, followed by degassing with argon for 15 min, and left at 70 °C for 4 h. The bPEG polymer was purified by precipitation in a mixture of diethyl ether and *n*-hexane (1:1) for 3 times and dried in vacuo.

bPEG polymers were also synthesized using BTPA as RAFT agent, and a second polymerization was conducted to incorporate different numbers of anchoring groups, obtaining tricarboxylic acid (triCA)-, pentaCA-, CA_48_-, triphosphonic acid (triPA)-, pentaPA-, and PA_55_-terminated bPEG polymers.

### Deprotection of bPEG Polymer

500 mg (0.02 mmol) of bPEG was dissolved in 3 mL of DCM under ice bath and sealed with septa. The mixture was degassed with argon for 10 min and cooled down. Then, 62.4 mg (0.40 mmol) of bromotrimethylsilane (TMSBr) dissolved in 2 mL of DCM was added dropwise to the reaction mixture. Then, the reaction was left at room temperature for 24 h. After complete reaction, DCM was removed by evaporation and 5 mL of methanol was added for another 4 h. The final diphosphonic acid-terminated bPEG polymer was obtained by precipitation in a mixture of diethyl ether and *n*-hexane (1:1) and drying in vacuo.

### Aminolysis of bPEG Polymer

Typically, 500 mg (0.02 mmol) of bPEG polymer was dissolved in 5 mL of THF. 500 μL of triethylamine and 36.57 mg of butylamine (0.5 mmol, 25-fold molar excess with respect to the trithiocarbonate moiety) were added to the mixture. The reaction was left at room temperature for 24 h. Thiol-terminated bPEG polymer was obtained after purification by precipitation using diethyl ether and *n*-hexane as precipitant and drying in vacuo.

### Preparation of Polymers/LMNPs

Typically, 50 mg of TTC-bPEG polymer and 58 mg of gallium–indium eutectic (EGaIn, Ga 75.5%/In 24.5%, Sigma-Aldrich) were added and dissolved in 5 mL of Milli-Q water precooled at ice bath for 30 min. Then the solution was sonicated under ice bath for 40 min (20% power, Sonics VCX-750 Vibra Cell Ultra Sonic Processor). After sonication, the mixture was centrifuged at 1,000 rpm for 10 min to remove the bulk aggregates. The supernatant was collected for further study, and the concentration of polymer/LMNPs was determined by weighing freeze-dried samples.

### Nuclear Magentic Resonance (NMR)

^1^H NMR spectra were obtained using a Bruker Avance 400 MHz spectrometer at 298 K.

### Size-Exclusion Chromatography (SEC)

The molecular weight (*M*_n_) and molar mass dispersity (*Đ* = *M*_w_/*M*_n_) of the polymers were determined by SEC using a Waters Alliance 2690 Separation Module equipped with a Waters 2414 differential refractive index (RI) detector, a Waters 2489 UV/visible detector, a Waters 717 Plus Autosampler, and a Waters 1515 Isocratic HPLC pump. THF or DMF was used as the mobile phase with a flow rate of 1 mL/min. The system was calibrated using polystyrene standards with molecular weights ranging from 6.82 × 10^2^ to 1.67 × 10^6^ g/mol.

### Dynamic Light Scattering

The hydrodynamic sizes of the polymers/LMNPs were analyzed at 298.0 K using Zetasizer Ultra (Malvern) equipped with a solid state He–Ne laser (λ = 633 nm).

### Transmission Electron Microscopy (TEM)

TEM images were obtained using a Hitachi HT7700 B equipped with a tungsten filament.

### Scanning Transmission Electron Microscopy (STEM) and Energy-Dispersive X-ray Spectroscopy (EDS) Mapping

STEM images and EDS mapping images were obtained using a Hitachi HF5000 Cs-STEM/TEM system equipped with Oxford EDX 2 x 1 sr 100 mm SDD EDX detectors.

### Fourier Transform Infrared (FT-IR) Spectra

FT-IR spectra were recorded on a Nicolet 6700 spectrometer under attenuated total reflectance (ATR). The spectra were collected over 128 scans with a spectral resolution of 10 cm^–1^.

## Supporting Information Available

Schematic illustration of the synthesis of brushed polymers (bPEG) with different anchoring moieties, ^1^H NMR and ^31^P NMR spectra of diphosphonate-CTA, ^1^H NMR spectra of TTC-bPEG, DiPA-bPEG, HS-bPEG, BTPA, PA monomer, BTPA-bPEG, CA-bPEG, and PA-bPEG, SEC traces of various polymers, and TEM images of CA_3_-bPEG/LMNPs, CA_5_-bPEG/LMNPs, CA_48_-bPEG/LMNPs, PA_3_-bPEG/LMNPs, PA_5_-bPEG/LMNPs, and PA_55_-bPEG/LMNPs at 0 and 48 h (PDF)
