# Supplementary material for: Engineering Polymers via Understanding the Effect of Anchoring Groups for Highly Stable Liquid Metal Nanoparticles
Source: ACS Appl Nano Mater. 2022 Feb 14;5(5):5959–71. doi: 10.1021/acsanm.1c04138 (PMC9150068; doi:10.1021/acsanm.1c04138)
Supplement: Supplementary file 1 — an1c04138_si_001.pdf [file an1c04138_si_001.pdf]

Supporting Information

Engineering Polymers via Understanding the Effect of Anchoring Groups for Highly Stable Liquid Metal Nanoparticles

Xumin Huang,[a,†] Tianhong Xu,[a,†] Ao Shen,[a] Thomas P. Davis,[a] Ruirui Qiao,[a,] and Shi-Yang Tang[b,*]*

[a]Australian Institute for Bioengineering and Nanotechnology, The University of Queensland, Brisbane, QLD 4072, Australia; Email: r.qiao@uq.edu.au

[b]Department of Electronic, Electrical and Systems Engineering, University of Birmingham, Edgbaston, Birmingham, B15 2TT, UK; Email: S.Tang@bham.ac.uk

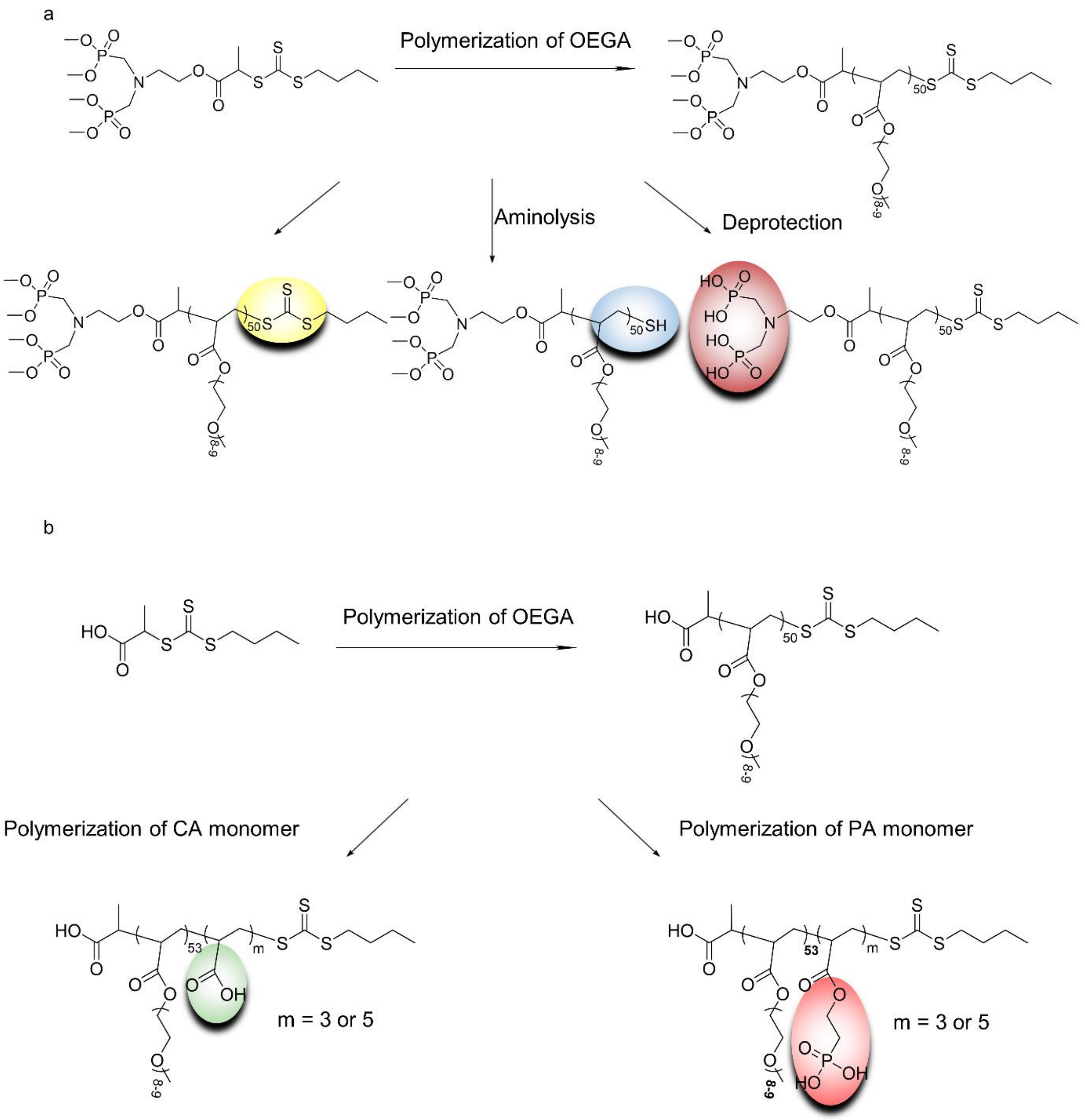

Figure S1. Schematic illustration of the synthesis of brushed polymers (bPEG) with different anchoring moieties.

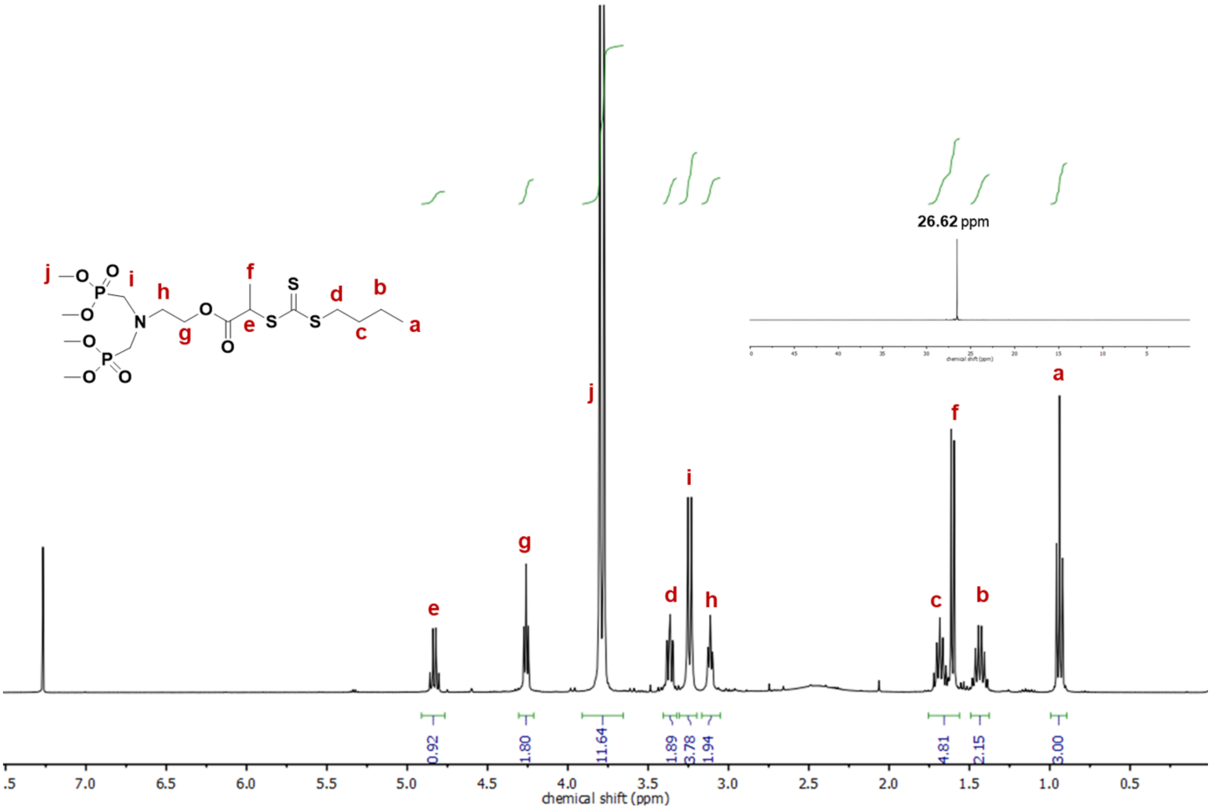

Figure S2. ^{1}H NMR and ^{31}P NMR spectra of diphosphonate-CTA.

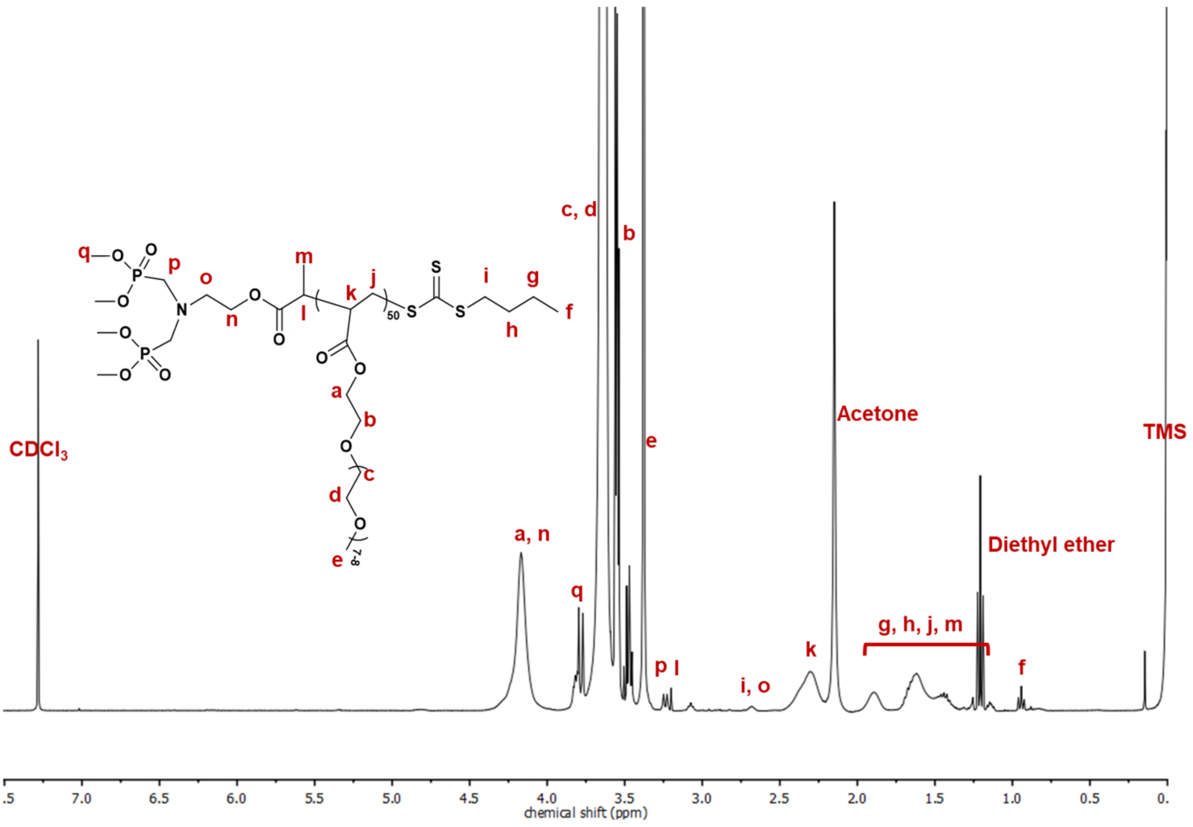

Figure S3. ^{1}H NMR spectrum of TTC-bPEG.

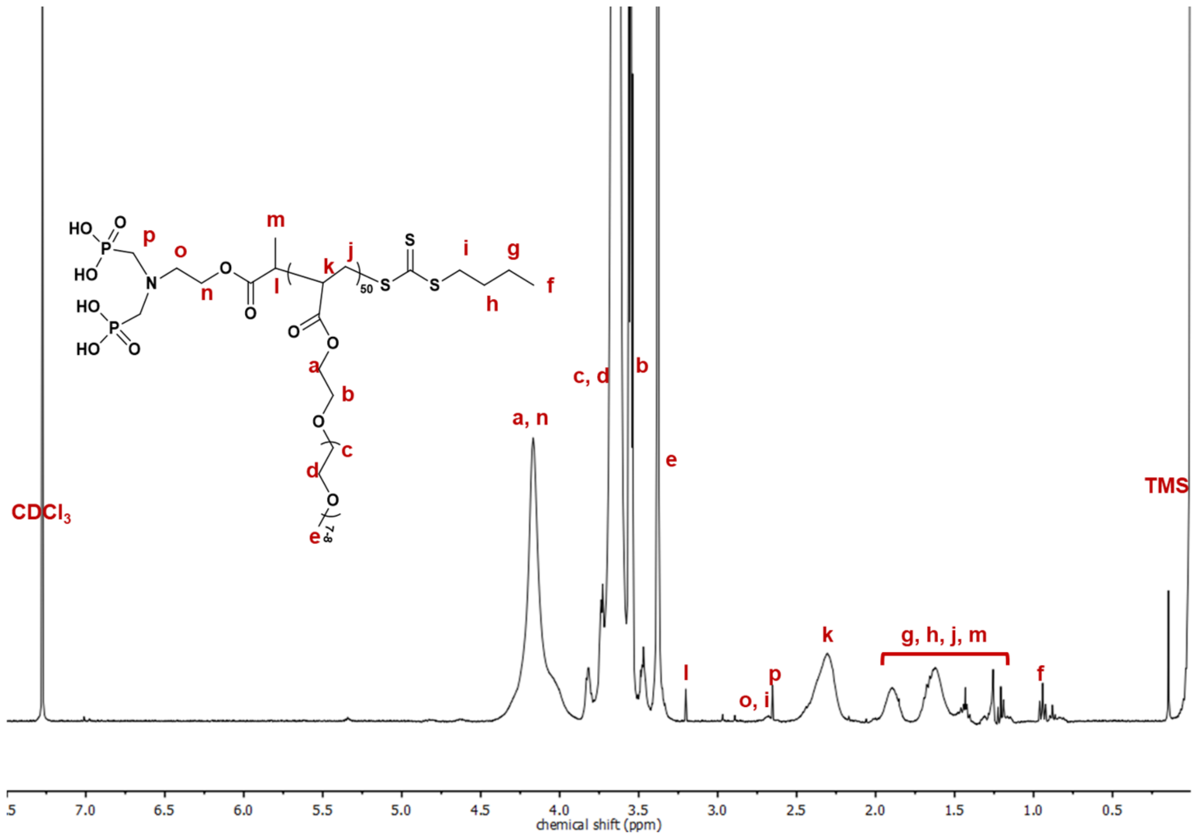

Figure S4. ^{1}H NMR spectrum of DiPA-bPEG.

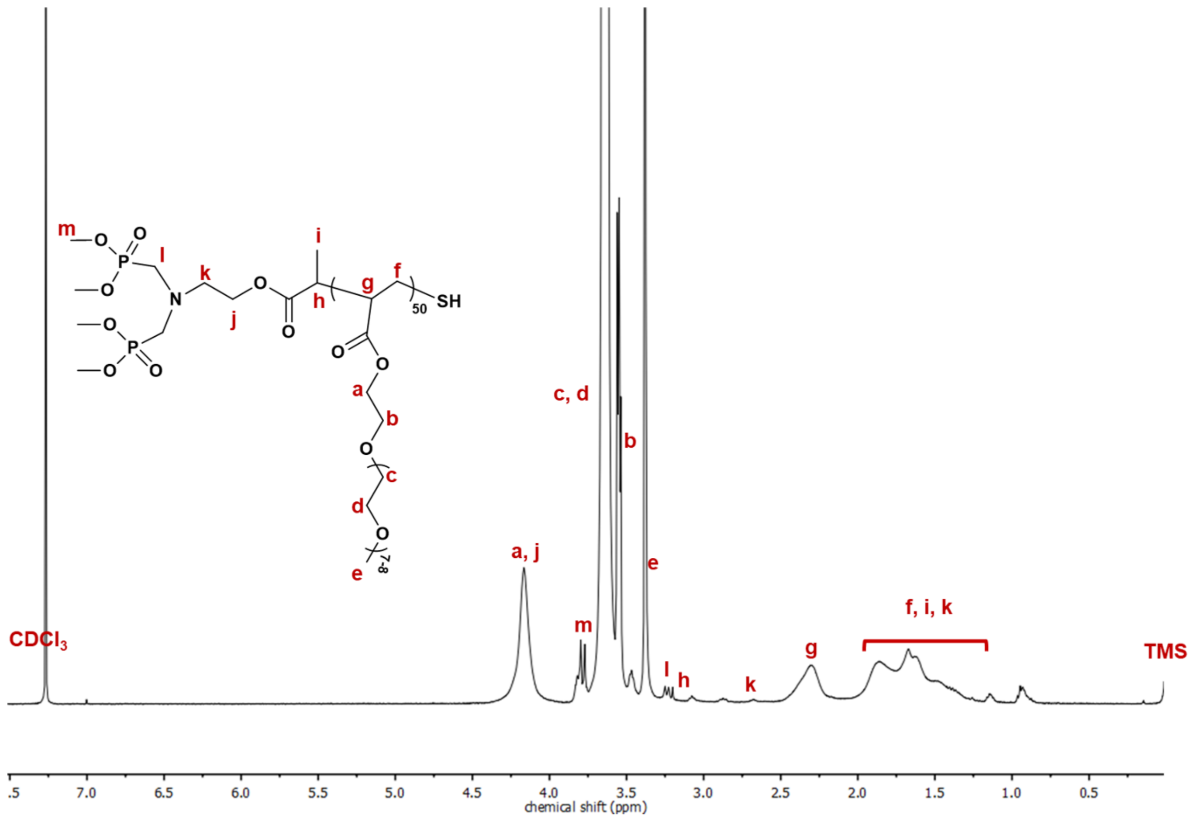

Figure S5. ^{1}H NMR spectrum of HS-bPEG.

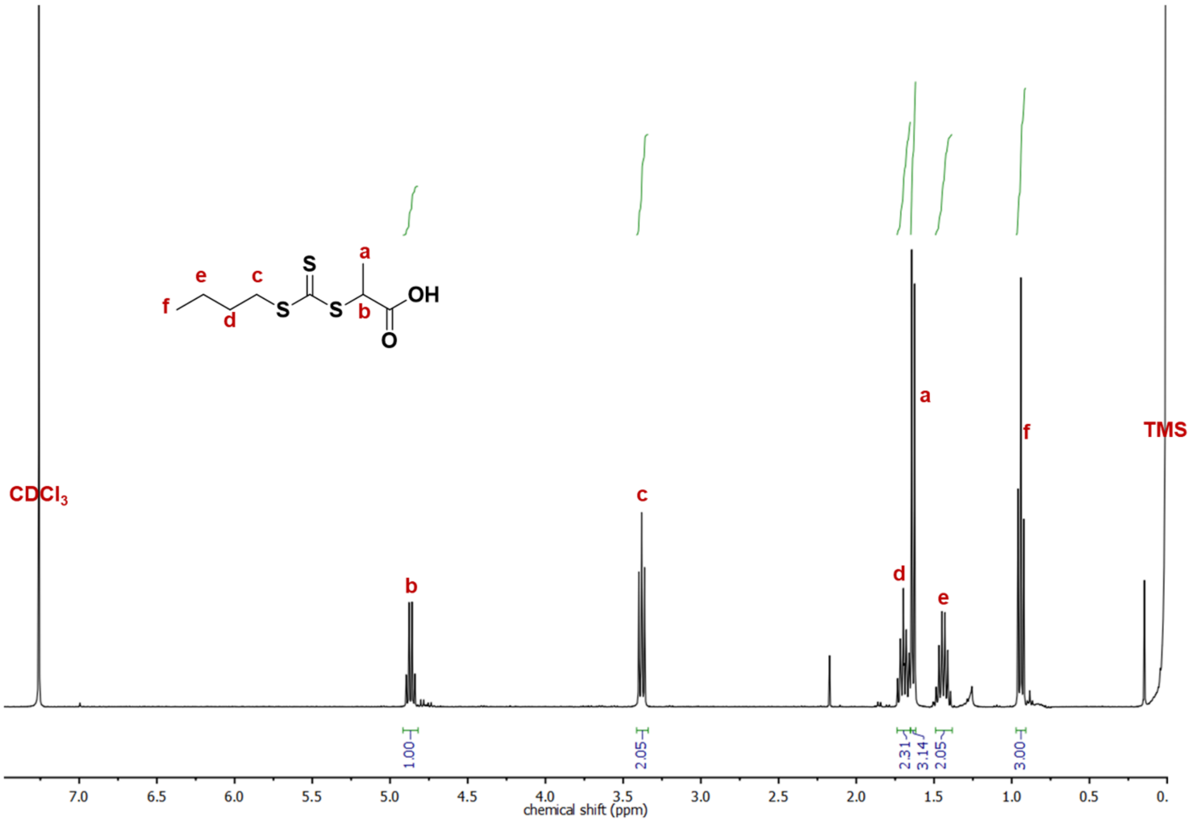

Figure S6. ^{1}H NMR spectrum of BTPA.

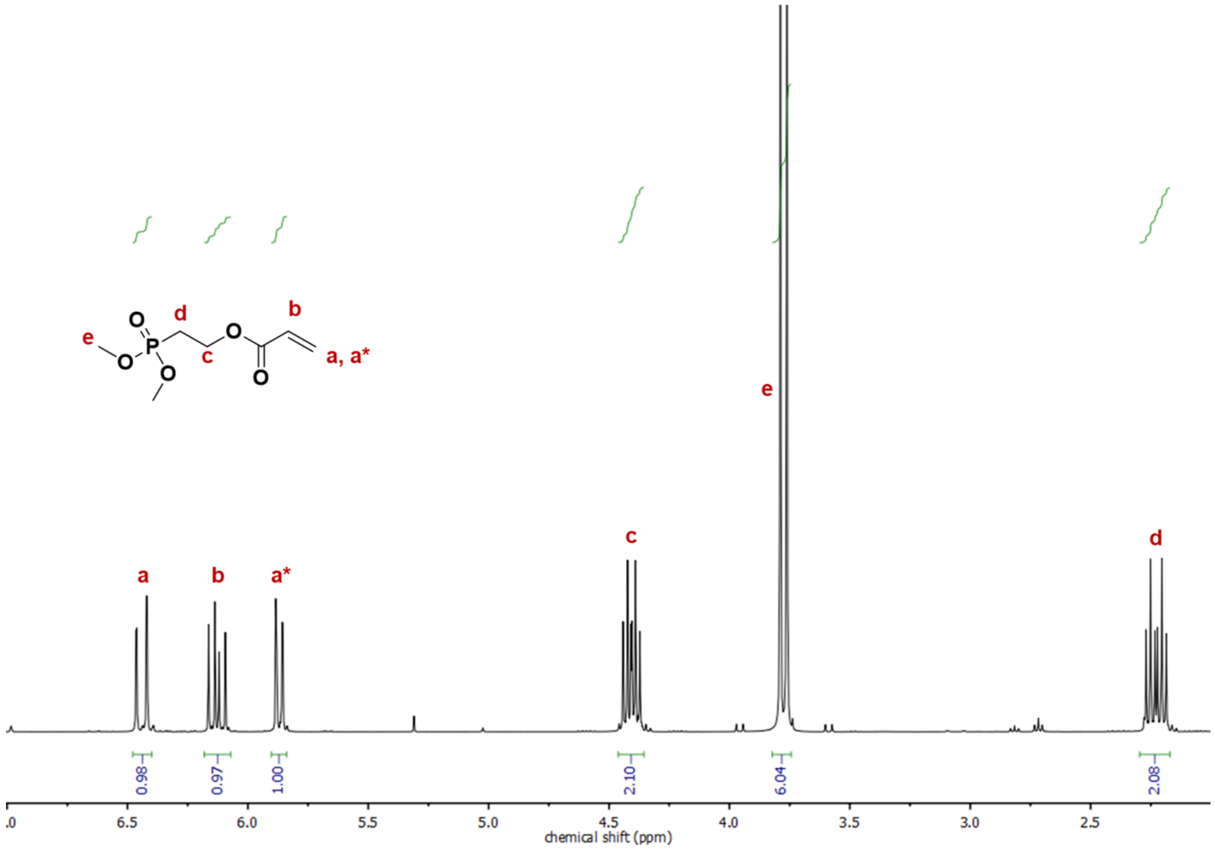

Figure S7. ^{1}H NMR spectrum of PA monomer.

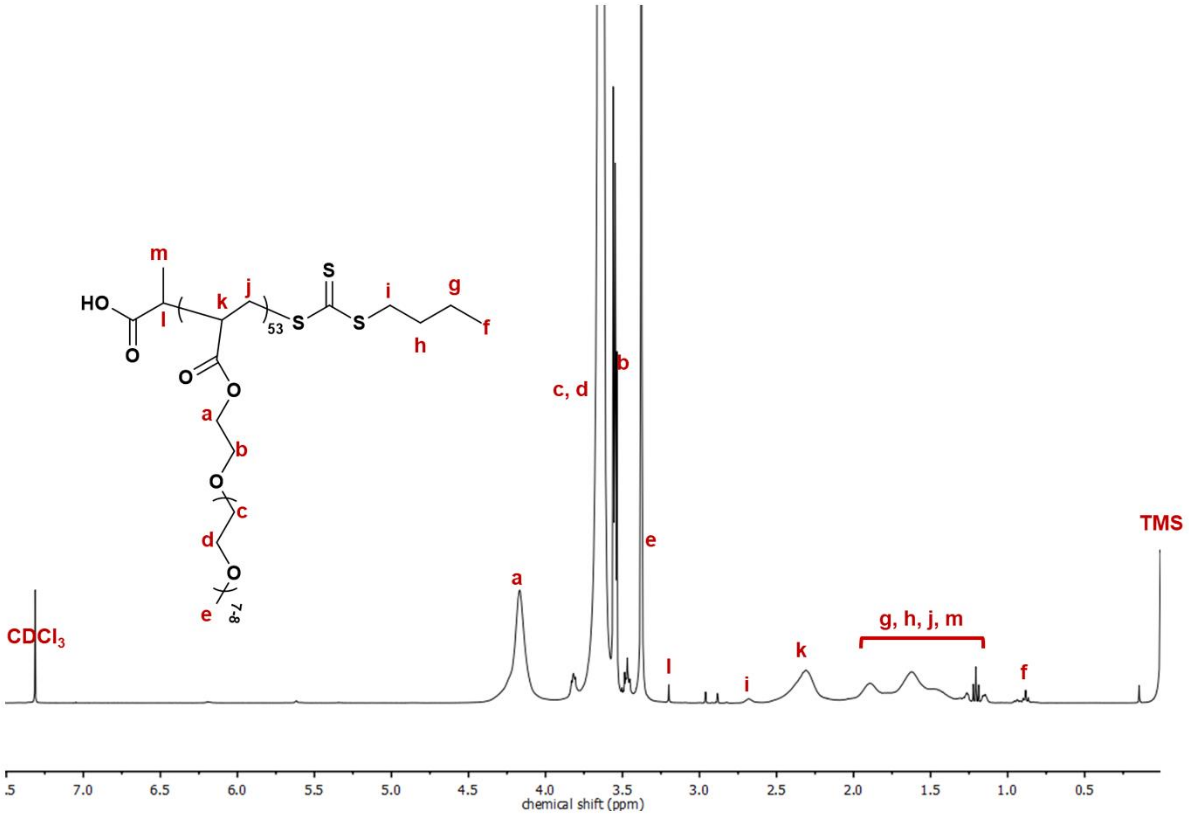

Figure S8. ^{1}H NMR spectrum of BTPA-bPEG.

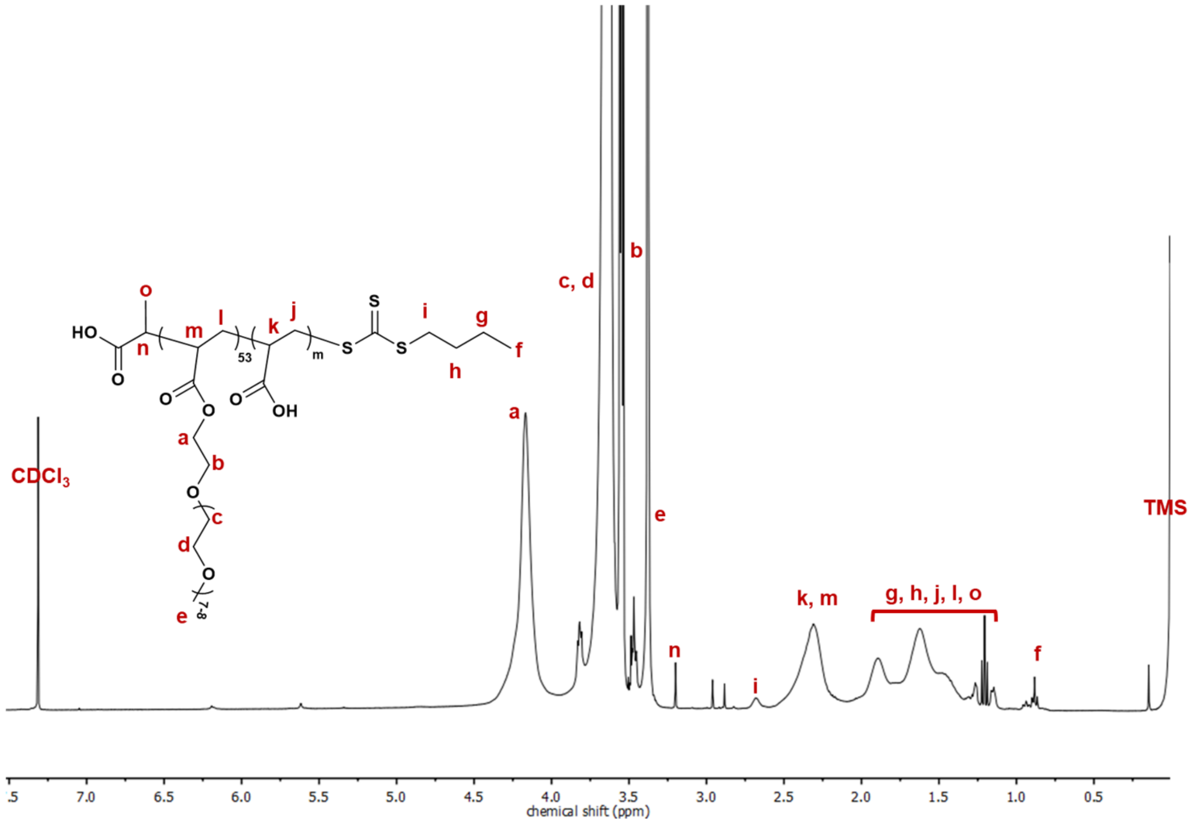

Figure S9. ^{1}H NMR spectrum of CA-bPEG.

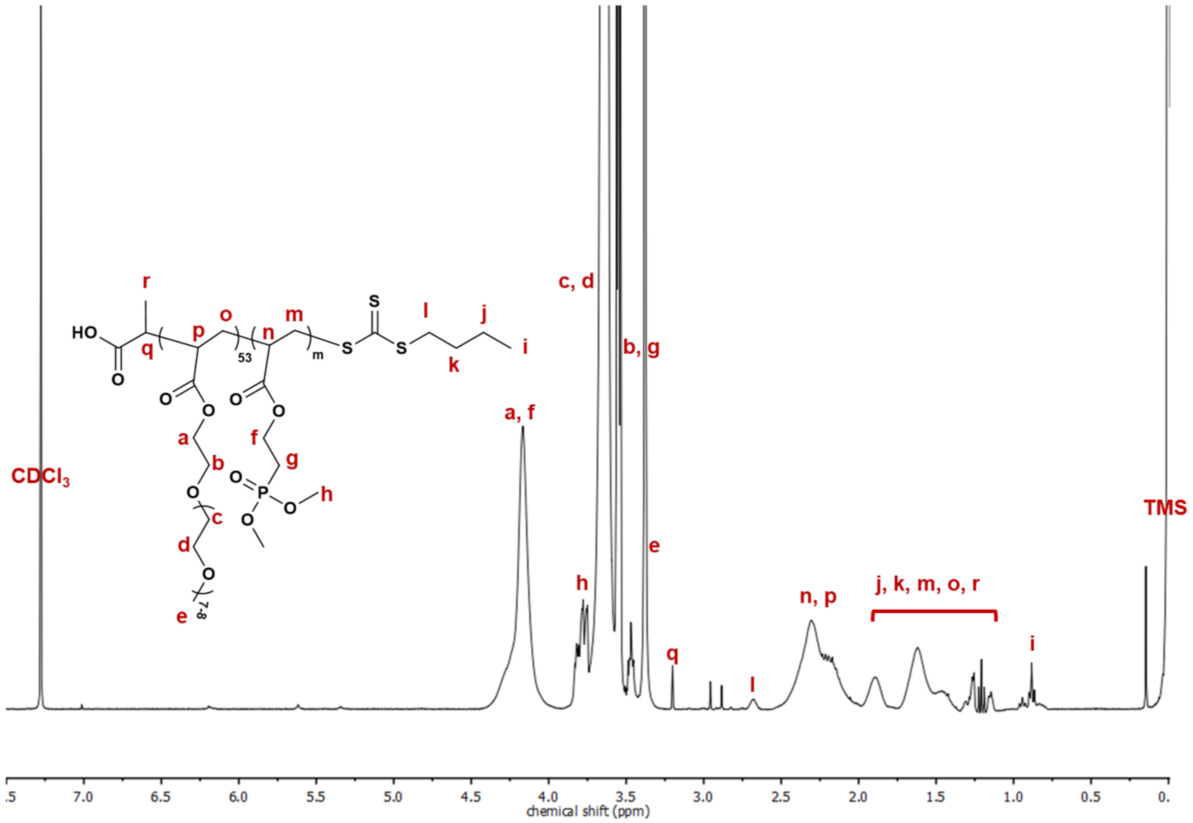

Figure S10. ^{1}H NMR spectrum of PA-bPEG.

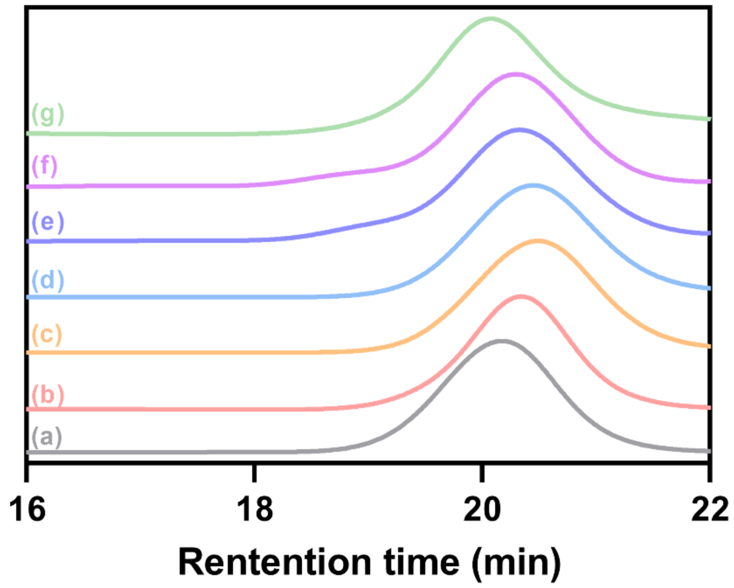

Figure S11. SEC traces of various polymer. (a)TTC-bPEG, (b)HS-bPEG, (c)CA_3-bPEG, (d) CA_5-bPEG, (e) PA_3-bPEG, (f) PA_5-bPEG, and (g)PA_{55}-bPEG.

Table S1. Summary of preparation of Polymers/LMNPs, stable duration in media, and chemical stability.

Name	Weight of EGaIn (mg)	Weight of Polymers (mg)	Recovery (%)	Stable duration in water	Stable duration in PBS	Stable duration in 0.9% NaCl	Stable duration in 10% FBS DMEM	Chemical stability after 48 h
TTC-bPEG/LMNPs	67	51	76.3	~ 48 h	< 3 h	~ 3 h	~ 24 h	No
HS-bPEG/LMNPs	59	53	62.5	< 3 h	< 3 h	~ 3 h	~ 24 h	Yes
DiPA-bPEG/LMNPs	52	49	64.4	~ 48 h	< 3 h	~ 48 h	~ 48 h	Yes
CA_3-bPEG/LMNPs	64	55	75.6	~ 48 h	< 3 h	< 3 h	~ 24 h	No
CA_5-bPEG/LMNPs	58	52	72.7	~ 48 h	< 3 h	< 3 h	~ 3 h	No
CA_{48}-bPEG/LMNPs	53	57	77.3	~ 7 days	~ 7 days	~ 7 days	~ 7 days	No
PA_3-bPEG/LMNPs	65	53	67.8	~ 48 h	< 3 h	~ 7 days	~ 3 h	Yes
PA_5-bPEG/LMNPs	62	52	61.4	~ 7 days	~ 7 days	~ 7 days	~ 3 h	Yes
PA_{55}-bPEG/LMNPs	55	55	59.1	~ 7 days	~ 7 days	~ 7 days	~ 7 days	Yes
Naked LMNPs	51		68.6	~ 24 h	< 3 h	< 3 h	~24 h	No

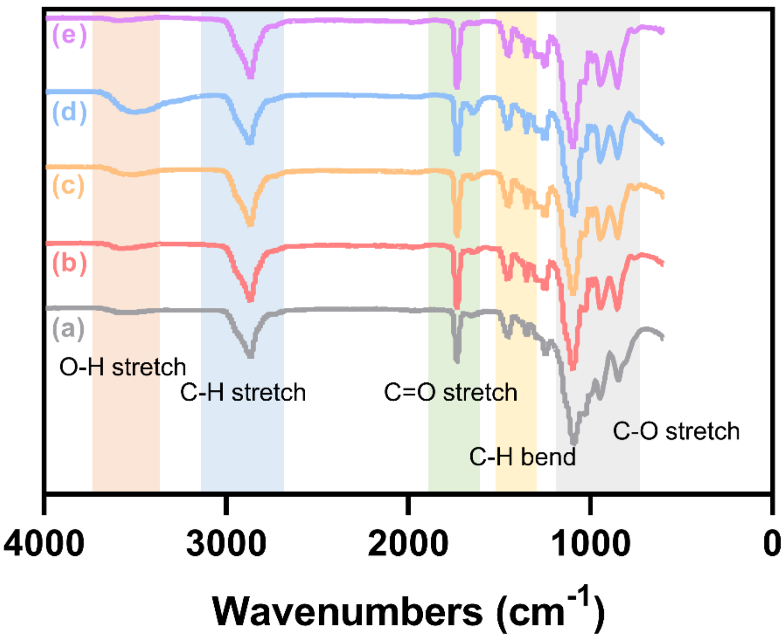

Figure S12. FT-IR spectra of synthesized polymers. (a)TTC-bPEG, (b)HS-bPEG, (c)DiPA-bPEG, (d) PA-bPEG, and (e) CA-bPEG

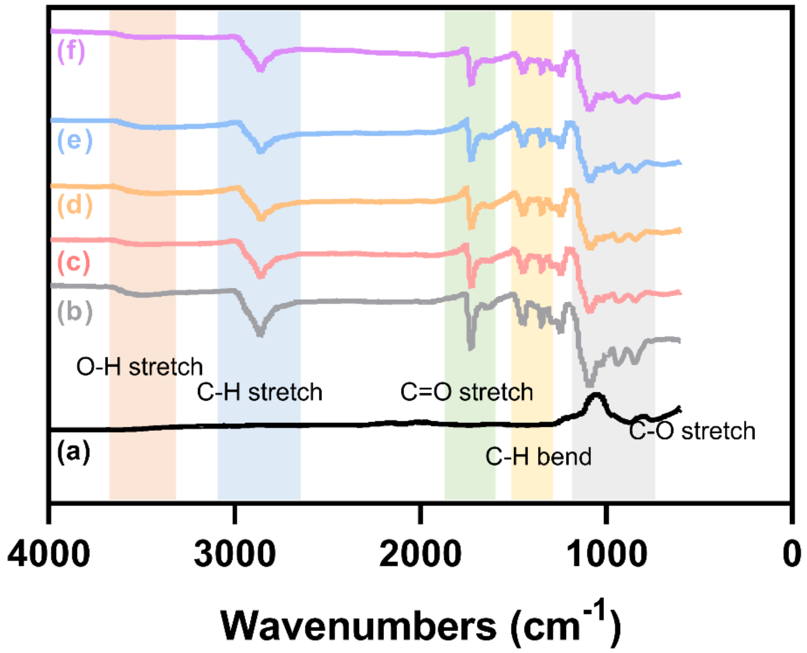

Figure S13. FT-IR spectra of Polymers/LMNPs. (a) TTC-bPEG/LMNPs, (b) HS-bPEG/LMNPs, (c) DiPA-bPEG/LMNPs, (d) PA-bPEG/LMNPs, and (e) CA-bPEG/LMNPs

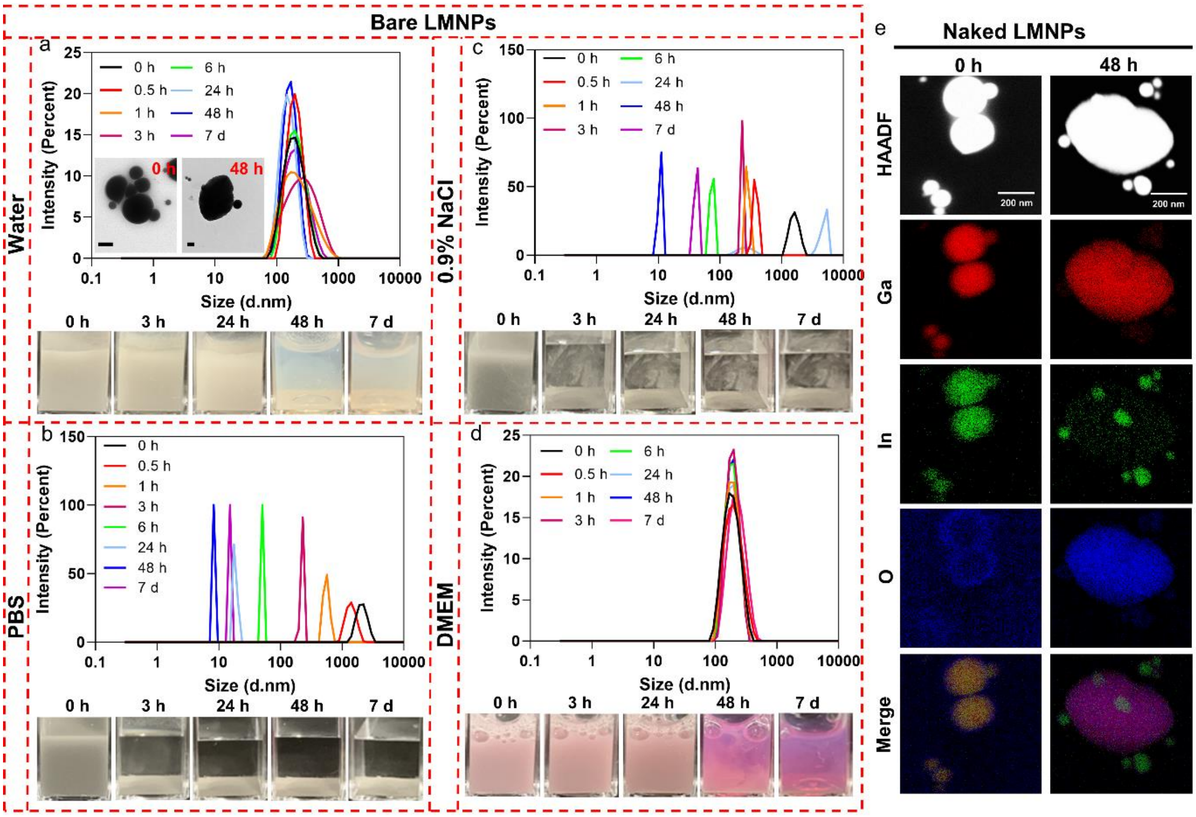

Figure S14. Evaluation of colloidal and chemical stability of bare LMNPs without polymer coating. Hydrodynamic size distributions and photographs of bare LMNPs in water (a), PBS (b), 0.9% NaCl (c), and 10% FBS DMEM culture medium (d) at different time points. Inset of (a) show TEM images of bare LMNPs at 0 h and 48 h. Scale bar are 100 nm.; (e) EDS mappings of bare LMNPs 0 h and 48 h after production in water.

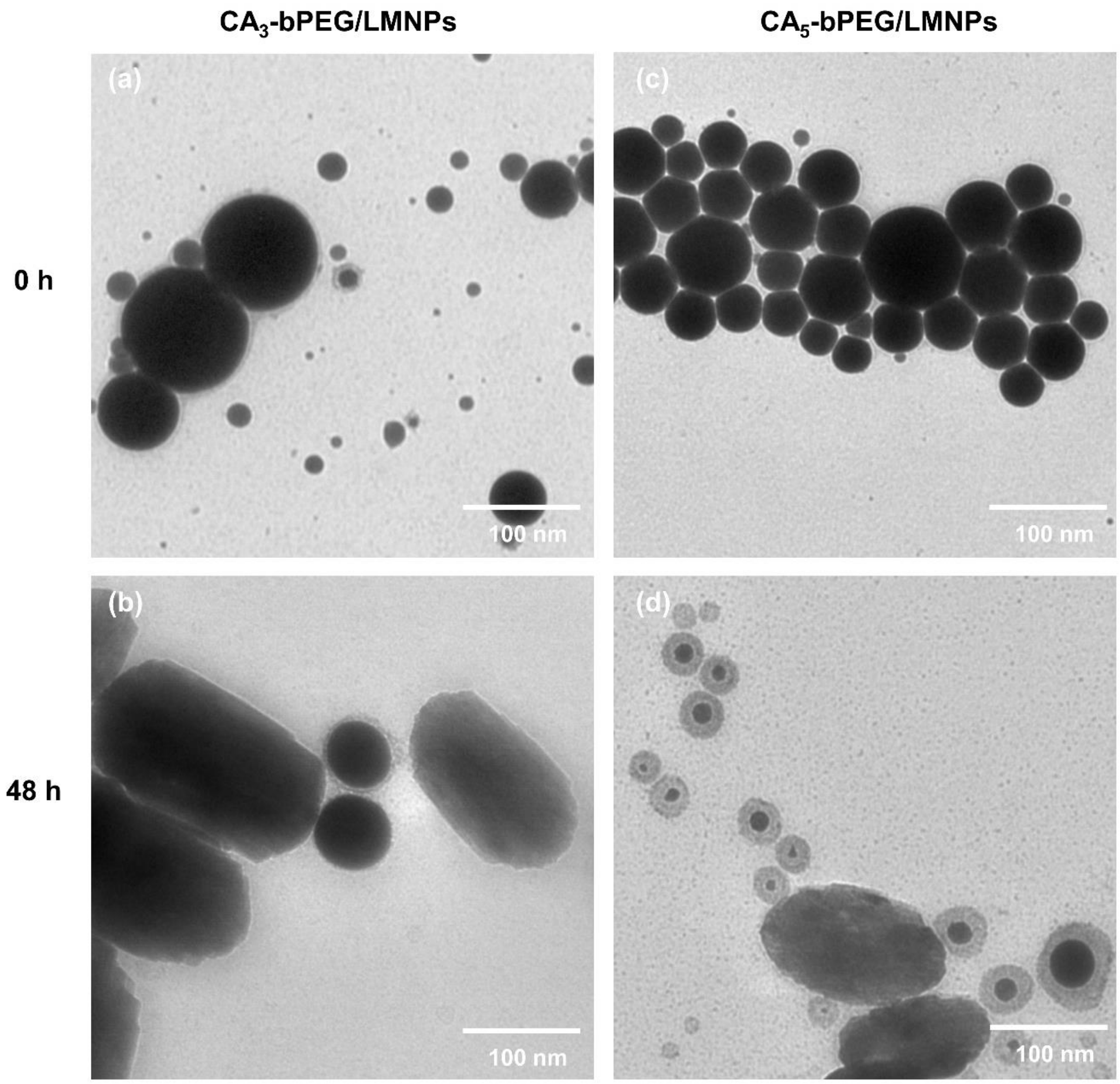

Figure S15. TEM images of CA$_3$-bPEG/LMNPs at 0 h (a) and 48 h (b) and CA$_5$-bPEG/LMNPs at 0 h (c) and 48 h (d).

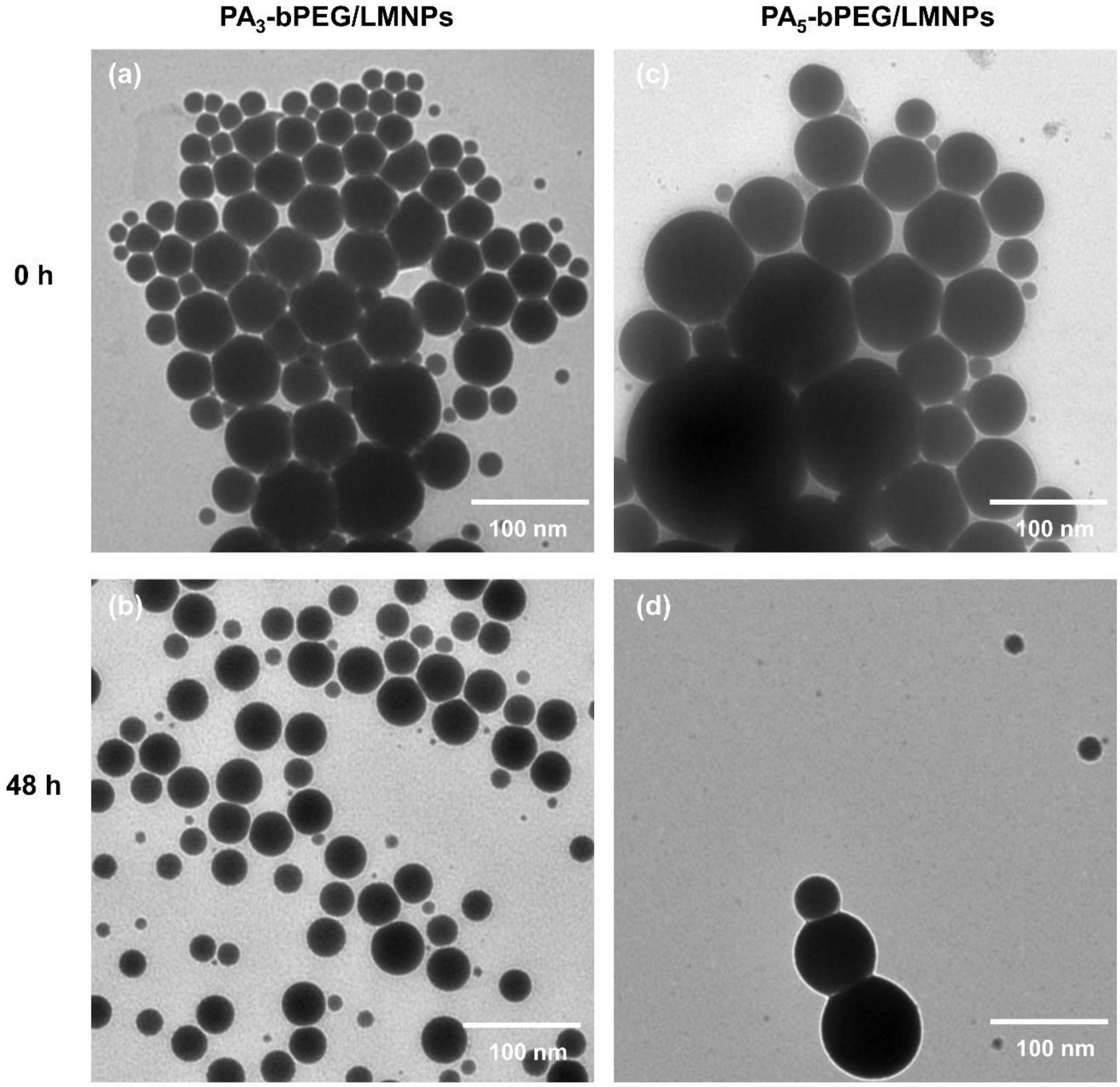

Figure S16. TEM images of PA_3-bPEG/LMNPs at 0 h (a) and 48 h (b) and PA_5-bPEG/LMNPs at 0 h (c) and 48 h (d).
